# Comparative landscape of genetic dependencies in human and chimpanzee stem cells

**DOI:** 10.1016/j.cell.2023.05.043

**Published:** 2023-06-20

**Authors:** Richard She, Tyler Fair, Nathan K. Schaefer, Reuben A. Saunders, Bryan J. Pavlovic, Jonathan S. Weissman, Alex A. Pollen

**Affiliations:** 1 Whitehead Institute for Biomedical Research, Cambridge, MA, USA; 2 Eli and Edythe Broad Center of Regeneration Medicine and Stem Cell Research, University of California, San Francisco, San Francisco, CA, USA.; 3 Biomedical Sciences Graduate Program, University of California, San Francisco, San Francisco, CA, USA; 4 Department of Cellular and Molecular Pharmacology, University of California at San Francisco, San Francisco, CA, USA.; 5 Department of Biology, Massachusetts Institute of Technology, Cambridge, MA, USA.; 6 Howard Hughes Medical Institute, Massachusetts Institute of Technology, Cambridge, MA, USA.; 7 Department of Neurology, University of California, San Francisco, San Francisco, CA, USA.; 8 David H. Koch Institute for Integrative Cancer Research, Massachusetts Institute Technology, Cambridge 02142, MA; 9 These authors contributed equally: Richard She, Tyler Fair.; 10 Lead contact

## Abstract

Comparative studies of great apes provide a window into our evolutionary past, but the extent and identity of cellular differences that emerged during hominin evolution remain largely unexplored. We established a comparative loss-of-function approach to evaluate whether changes in human cells altered requirements for essential genes. By performing genome-wide CRISPR interference screens in human and chimpanzee pluripotent stem cells, we identified 75 genes with species-specific effects on cellular proliferation. These genes comprised coherent processes, including cell cycle progression and lysosomal signaling, which we determined to be human-derived by comparison with orangutan cells. Human-specific robustness to *CDK2* and *CCNE1* depletion persisted in neural progenitor cells and cerebral organoids, supporting the G1-phase length hypothesis as a potential evolutionary mechanism in human brain expansion. Our findings demonstrate that evolutionary changes in human cells reshaped the landscape of essential genes and establish a platform for systematically uncovering latent cellular and molecular differences between species.

## Introduction:

Comparative studies of humans and chimpanzees, our closest extant relatives, have long sought to define the evolutionary origins of unique human features. Within seven million years, humans evolved numerous specializations, from bipedalism to the threefold expansion of the cerebral cortex^1,2^. Many of these novel human traits emerge from changes in cell behavior during development. These changes in cell behavior may in turn reflect underlying differences in molecular circuits that emerged during the short time scale of hominin evolution. However, we currently lack a framework for systematically identifying which molecular pathways play divergent roles in conserved developmental cell types.

Current approaches for studying the molecular basis of human evolution include reconstructing candidate mutations at specific loci in model organisms, but only a handful of mutations in non-coding regulatory regions and coding genes have been examined in detail. Among conserved non-coding elements with unexpected changes in the human lineage, specific loci have been linked to gene expression changes in distal limbs^3^, increased sweat gland number^4^, and increased neural proliferation^5^. Among coding changes, two human-specific coding mutations in *FOXP2* have been proposed to contribute to human language capabilities based on functional studies in mouse models and human genetics^6,7^, and three modern human-specific mutations in *KIF18A* and *KNL1* prolong metaphase and reduce segregation errors in neural progenitor cells^8^. In addition, recent duplications and subsequent modifications of *ARHGAP11B* and *NOTCH2NL* have been implicated in the expansion of the human cortex^9-12^, supporting predictions that human-specific mutations may influence proliferation of neural progenitor cells during development^13,14^. Nonetheless, connecting individual candidate mutations to evolved human traits remains challenging because most mutations are neutral or low effect size, analyses are low throughput, and we lack a detailed understanding of the divergence in cellular and developmental phenotypes that ultimately give rise to species differences.

In parallel, high-throughput genomics-based approaches have described gene regulatory changes that may contribute to species differences. Because ape primary tissue is largely inaccessible during early development, recent studies have employed stem cell derived models as an experimentally tractable system for comparative analyses of species differences during development. Thousands of cell type-specific gene expression differences have been identified in pluripotent stem cells^15,16^, cardiomyocytes^17^, endoderm^18^, neural crest^19^, and cortical neurons^20-22^. However, these gene expression differences comprise a mixture of neutral changes, causal changes, and indirect downstream consequences, and genes that mediate species differences may have conserved expression. Therefore, it can be difficult to ascertain which molecular changes, among hundreds or thousands, drive differences in cellular physiology.

The history of developmental genetics provides a template for linking the function of individual genes to organismal phenotypes. Early mutagenesis screens in *Drosophila melanogaster* identified genes critical for body axis patterning^23,24^. Many of these genes belonged to highly conserved cell signaling pathways that also coordinate development in vertebrates, such as Wnt^25,26^, Hedgehog^27^, and BMP^28^. More recent efforts in organismal screening involve several international consortia that have generated large collections of knockout mice to investigate more complex vertebrate phenotypes^29-31^. The success of these genetic approaches has resulted in the functional annotation of many of the genes that guide mammalian development. However, despite the conservation of many core developmental principles from fruit flies to mice to humans, these shared molecular functions do not account for how our species evolved to be different.

To apply functional genomics approaches to questions of species divergence, we harnessed recent advances in CRISPR-based technologies that have enabled genome-scale perturbation screens across thousands of human cell lines^32-34^. These efforts have mapped landscapes of genetic dependencies with an enrichment of essential genes in coherent pathways that typically cluster by cell type of origin^35,36^. Extending this approach to studies of comparative evolution could reveal genes or cellular processes with divergent functional roles in homologous cell types. Illuminating the extent and identity of recently evolved genetic dependencies would complement individual candidate gene approaches, descriptive comparative genomics analyses, and single species loss-of-function studies. However, whether genetic dependencies diverged in closely related hominin species and how this knowledge could reveal previously unappreciated differences in cellular physiology remains unexplored.

To evaluate the extent of conservation and divergence in genetic dependencies between human and chimpanzee, we established a comparative loss-of-function screening approach in pluripotent stem cells (PSCs). PSCs are a model for the earliest stages of development, capturing features of the inner cell mass of the blastocyst, including the capacity to differentiate into all germ layers at a stage that precedes species differences in developmental timing and cell type composition. The state of pluripotency is well conserved between human and chimpanzee PSCs at the level of the transcriptome, epigenome, and cell fate potential^15^, providing a homologous cell type for species comparison. In addition, PSCs have greater levels of open chromatin and gene expression than somatic cells^37^, enabling large-scale study of gene function for genes later expressed in diverse cell types. As PSCs are poised to self-renew or differentiate into all germ layers based on environmental cues, we reasoned that changes in proliferation in PSCs could provide a sensitive measure for species-specific responses to a wide range of genetic perturbations.

Performing genetic screens using an *in vitro* model confers several advantages that could support isolation of molecular and cellular species differences. First, the ability to grow large numbers of PSCs enables a pooled library approach with multiple redundant library elements targeting each gene. Second, laboratory cell culture provides a well-defined and highly controlled environment, which minimizes extrinsic sources of variation. Lastly, the scalability of pooled screening allows for retesting of each cellular phenotype in PSCs derived from multiple individuals of each species to account for individual variation within a species. Thus, we conducted genome-wide CRISPR-interference (CRISPRi) screens in human and chimpanzee PSCs. Despite high levels of conservation, our screens revealed that genetic dependencies can diverge in short evolutionary time scales, that species differences are organized into coherent pathways and protein complexes, and that human-specific changes have evolved in gene networks promoting G1/S progression in PSCs and neural progenitor cells. In addition to these specific insights, our study establishes a broadly applicable experimental approach for uncovering latent molecular differences between closely related species.

## Genome-wide CRISPRi screening in human and chimpanzee stem cells

**Figure 1.**
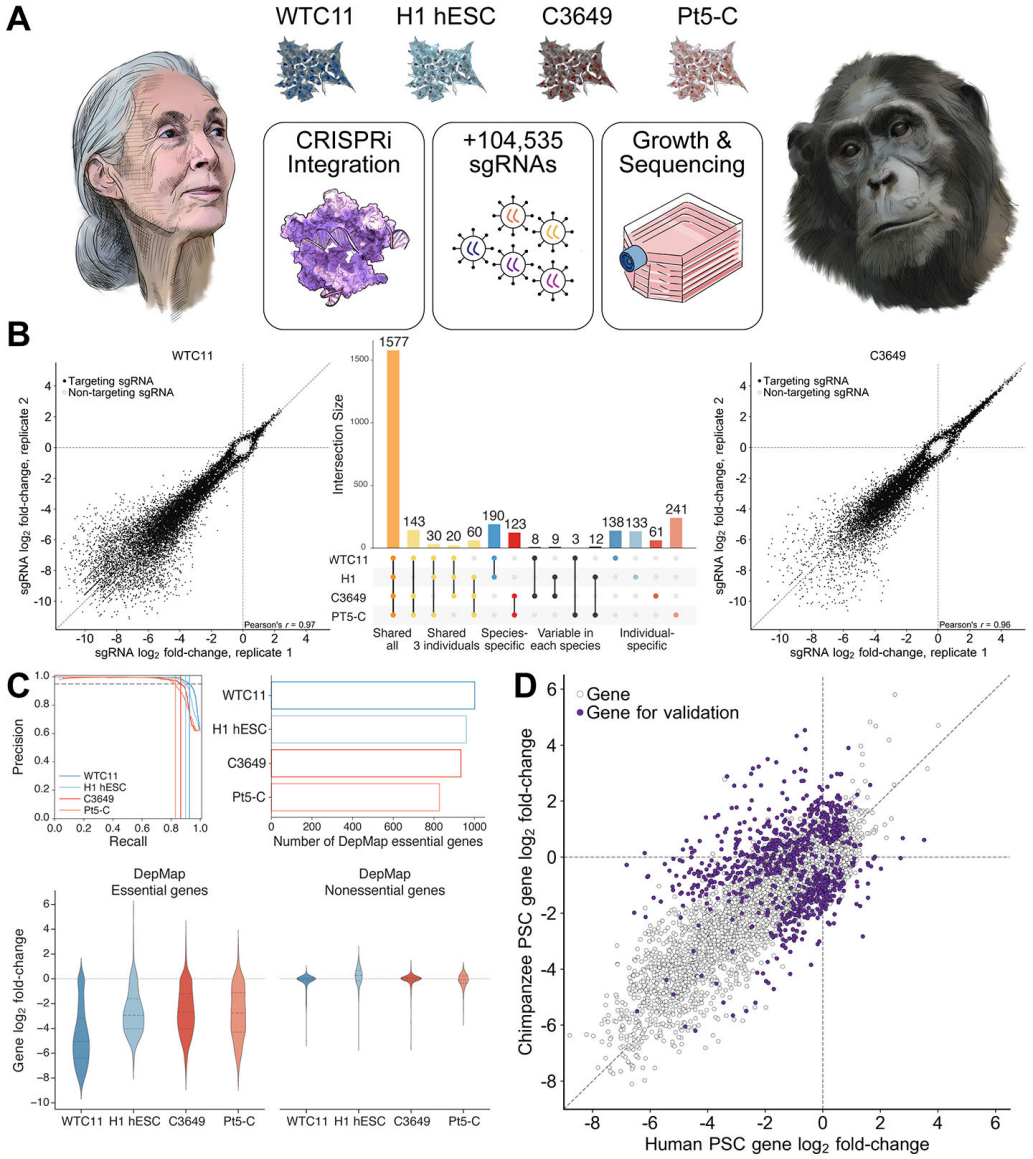
Genome-wide CRISPRi screens in human and chimpanzee stem cells identify candidate species-specific genetic dependencies (A) Schematic of CRISPRi screening approach, with original portraits of Jane Goodall and an adult female chimpanzee from Kibale Chimpanzee Project for artistic representation of human and chimpanzee species differences. Two human (WTC11 and H1) and two chimpanzee (C3649 and Pt5-C) PSC lines were engineered to express dCas9-KRAB, infected with the lentiviral hCRISPRi-v2 sgRNA library, and grown competitively for 10 days. Depleted and enriched sgRNAs were detected by high-throughput sequencing. (B) Scatterplots of sgRNA log_2_ fold-change for WTC11 and C3649 technical replicates and UpSet plot showing the intersection of essential genes across all four screens. (C) Precision-recall analysis (top left) for each screen. Precision and recall were determined using DepMap essential and nonessential genes. The number of DepMap essential genes (top right) identified by MAGeCK (5% FDR, log_2_ fold-change < −1.5). Distribution of log_2_ fold-change for DepMap essential (bottom left) and nonessential (bottom right) genes. (D) Species-level gene log_2_ fold-change across genome-wide CRISPRi screens. Gene-level phenotypes were computed as the mean of the three sgRNAs with the largest absolute log_2_ fold-change. sgRNAs lacking perfect-match targets in the chimpanzee genome were excluded from analysis. See also Figure S1 and Tables S1-S3.

To enable comparative CRISPR-based genetic screening, we engineered CRISPRi machinery^32^ at the *CLYBL* safe harbor locus^38^ in two human and two chimpanzee pluripotent stem cell lines (Figure 1A). For the two human individuals, we chose two well-characterized cell lines, WTC11^39-41^, an induced pluripotent stem cell (iPSC) line, and H1^42^, an embryonic stem cell (ESC) line. For the chimpanzee individuals, we chose two iPSC lines used in previous studies: C3649 and Pt5-C^15,43^ (Table S1).

To identify genes that modify cellular growth and survival, we infected each cell line with the genome-wide lentiviral hCRISPRi-v2 sgRNA library^44^ (5 sgRNAs/gene), selected for sgRNA-expressing cells with puromycin, cultured cells for 10 days, and quantified sgRNA enrichment and depletion by high-throughput sequencing (Table S2A). While hCRISPRi-v2 was designed to target the human genome, 77.4% of sgRNAs perfectly matched targets in the chimpanzee reference genome (panTro6); sgRNAs with mismatches were not considered for analyses of species differences (Figure S1A, STAR methods). Across all four screens, we observed robust depletion of sgRNAs targeting common essential genes and enrichment of sgRNAs targeting proliferation-suppressor genes. Analysis of technical and biological replicates revealed strong sgRNA correlations for replicates of the same cell line (Pearson’s *r* = 0.80 to 0.97, Figure 1B) and for different cell lines within species (*r* = 0.69 to 0.83). In addition, all four genetic screens sensitively and precisely distinguished Dependency Map (DepMap) common essential and nonessential genes^45,46^, recalling 82.9% to 92.6% of common essential genes at 95% precision (Figure 1C).

We next sought to identify genes with species-specific effects on cellular proliferation. To do so, we utilized MAGeCK^47^ and developed a bootstrapping-based method that accounted for both the number of significantly enriched or depleted sgRNAs targeting a gene and the magnitude of sgRNA log_2_ fold-change (Figures S1B, Tables S2B and S2C, STAR methods). While the large majority of essential genes were shared between species (Figure 1B), we identified 583 candidate species-specific essential genes and 202 candidate species-specific proliferation-suppressor genes (Figure 1D and Figure S1C). Importantly, this approach identified far fewer candidate genes exclusively shared between one individual of each species (*n* = 3 to 12 genes, Figure 1B), highlighting the influence of species on gene essentiality. As an additional quality control, we confirmed that sgRNAs targeting regulators of apoptosis such as *BAK1* and *BAD* were enriched in all four PSCs (Figure S1D). We unexpectedly discovered that sgRNAs targeting *TP53* were enriched only in the two human cell lines, suggesting that the two chimpanzee PSC lines used in primary screening were p53-unresponsive. We reasoned that a fraction of the candidate species differences might thus be attributable to p53 status and would require additional testing with p53-responsive chimpanzee cell lines (Figures S1E and S1F). In conclusion, our approach shows that genome-wide CRISPRi screening can be applied to closely related species to establish a comparative essential-ome and to nominate candidate species differences.

## Human and chimpanzee can be distinguished by genetic dependencies

We next sought to validate candidate species differences across multiple independently-derived human and chimpanzee PSCs to distinguish species differences from those driven by individual variation^48-50^, adaptation to cell culture^51^, or somatic cell reprogramming^52^. We engineered new CRISPRi stem cell lines from four human (H20961B, H21792A, H23555A, H28126B) and four chimpanzee (C3624K, C8861G, C40280L, C40290F) individuals. To minimize technical variation, we selected high quality cell lines with normal karyotypes that were reprogrammed with identical protocols (Table S1) and maintained in identical media. Cell lines from both species were previously shown to differentiate into all three germ layers via teratoma formation and embryoid body assays, functionally validating pluripotency^15^. Finally, the human and chimpanzee lines were previously shown to share comparable pluripotency scores with strongly overlapping patterns of H3K27me3 and H3K27ac at pluripotency genes^15^ and similar transcriptional trajectories of differentiation^18^, suggesting that the cell lines were in a comparable state of pluripotency. We used bulk RNA-seq to verify that the CRISPRi-engineered cell lines exhibited comparably high expression levels of canonical pluripotency markers *OCT4*, *SOX2*, and *NANOG*, compared to the original source lines (Figures S2A and S2B). No significant expression differences were observed for these genes in our human and chimpanzee engineered cell lines (*P* = 0.42, 0.98, and 0.94, two-tailed t-test). We analyzed copy number variation using CaSpER^53^ and genome sequencing coverage to rule out the presence of large duplications or deletions (Figure S2C). In addition, we assessed CRISPRi cell lines for p53-responsiveness by measuring sensitivity to nutlin-3a, a small molecule MDM2 inhibitor that induces p53-dependent autophagy and apoptosis^54,55^. All 11 new human and chimpanzee lines plus WTC11 and H1 were MDM2/p53-responsive, while C3649 and Pt5-C, the chimpanzee lines used for genome-scale screening, were nonresponsive to MDM2/p53 perturbations (Figures S1E and S1F).

**Figure 2.**
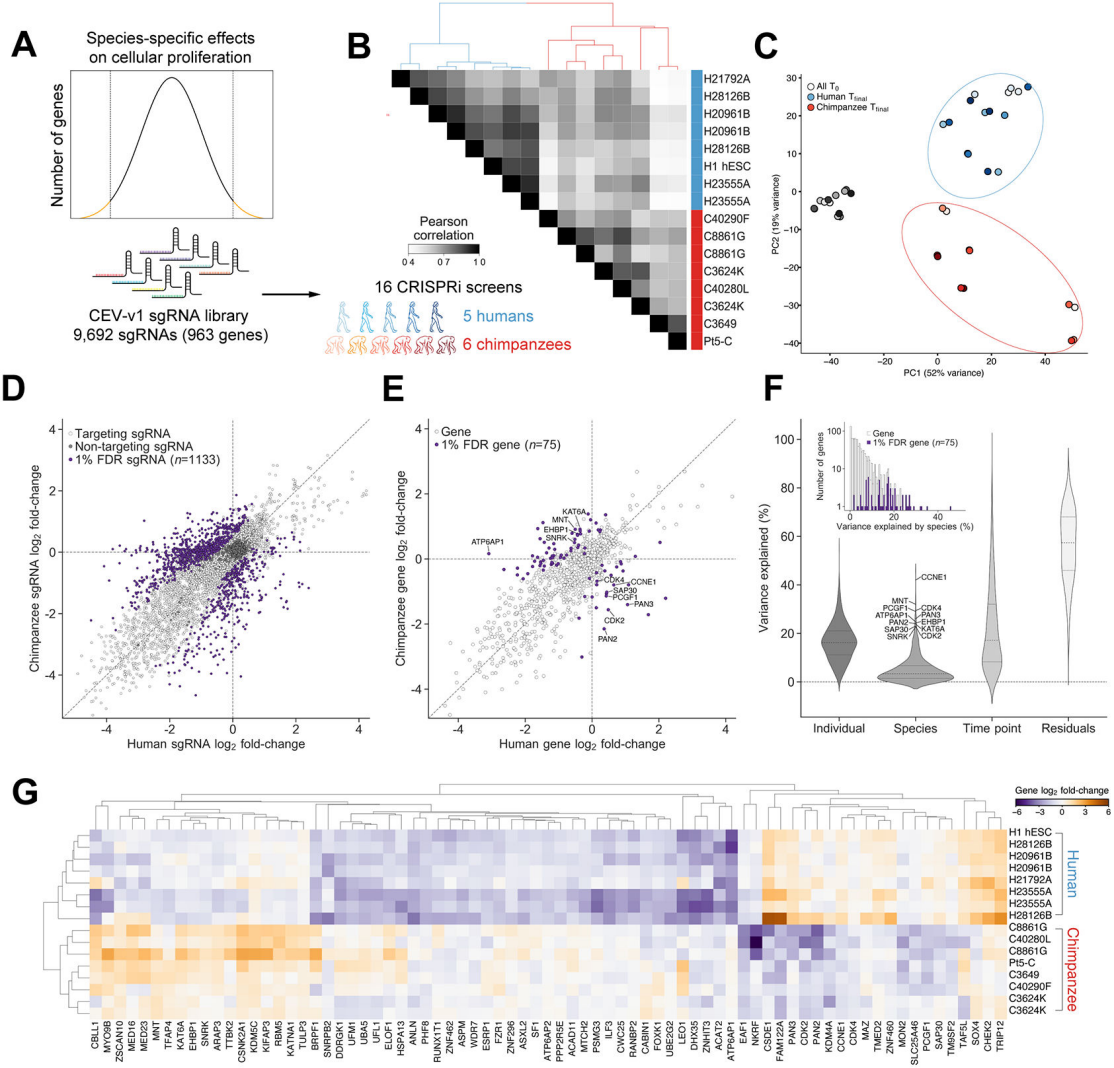
Species-specific genetic dependencies validate across five human and six chimpanzee individuals (A) Schematic of validation sgRNA library design and CRISPRi screening approach. (B) Heatmap of Pearson correlations and hierarchical clustering for sgRNA profiles across 16 validation CRISPRi screens. Individuals listed twice are replicate screens performed in separate laboratories. (C) Principal component analysis of sgRNA counts at t_0_ (black circle) and t_final_ (red and blue circles). (D) Scatterplot of log_2_ fold-change of sgRNA counts, modeled by DESeq2. 1,133 sgRNAs with significant species differences (FDR < 0.01) colored in purple and negative-control sgRNAs colored in dark gray. (E) Species-level gene log_2_ fold-change across validation CRISPRi screens. Gene-level phenotypes were computed as the mean of the four sgRNAs with the largest absolute log_2_ fold-change. The 12 genes with the greatest variance in sgRNA log_2_ fold-change attributable to species are labeled. (F) Dream-variancePartition analysis for quantifying sources of variation in sgRNA counts attributable to individual, species, and timepoint (t_0_ vs. t_final_). (G) Heatmap of gene log_2_ fold-change and hierarchical clustering for 75 genes with species-specific effects on cellular proliferation across validation CRISPRi screens (1% FDR). See also Figures S2, S3, and Tables S4-S5.

To enable secondary screening, we designed a comparative essential validation (CEV-v1) library consisting of 7,847 sgRNAs targeting the transcriptional start sites of 963 genes from our genome-scale datasets (8 sgRNAs per gene, STAR methods) and 1,845 negative-control sgRNAs (Figure 2A; Figures S2D-G, Table S4A-C). Due to the scalability of pooled screening, we targeted an inclusive set of genes with significant or suggestive differences between species in the primary screens as well as gene families with notable evolutionary histories^56,57^. To reduce human-specific bias, we required that every sgRNA in CEV-v1 perfectly match target sites in the human (hg38) and chimpanzee (panTro6) reference genomes^58^. In total, we performed 16 CRISPRi screens using the CEV-v1 sgRNA library (Figure 2A, Table S4A-C). The validation screens were performed in the four newly constructed CRIPSRi PSC lines of each species. In addition, we retested three of the four PSC lines used for genome-scale screening (Pearson's *r* = 0.76 to 0.89) and performed biological replicate screens in separate laboratories for five cell lines (3 human lines, 2 chimpanzee lines, *r* = 0.70 to 0.92). Notably, hierarchical clustering of cell lines by the similarity of their sgRNA profiles separated all the human (including ESCs and iPSCs) from all the chimpanzee individuals (Figure 2B). Decomposition of each cell line’s sgRNA profile by principal component analysis also grouped individuals by species, with the main axes of variation relating to shared changes in sgRNA representation over time (PC1) and species-specific changes (PC2) (Figure 2C). Together, our findings show that stem cells from humans and chimpanzees can be distinguished by their responses to genetic perturbations.

## Molecular nature of core species-specific genetic dependencies

We next sought to identify genes underlying the differences between human and chimpanzee sgRNA profiles. We used DE-seq2^59^ to model sgRNA counts from all human and chimpanzee CEV-v1 screens and contrasted the species terms to detect sgRNAs with divergent effects on cellular proliferation (STAR methods). We identified 1,133 sgRNAs with evidence for differences between species (1% false discovery rate (FDR), ∣chimpanzee–human log_2_ fold-change∣ ≥ 0.5), while negative-control sgRNAs were tightly distributed around zero (Figure 2D). Using α-RRA^47^ to combine sgRNA *P*-values, we found 75 genes with robust species-specific effects on cellular proliferation at a 1% FDR (Figures 2E-G; Figure S3; STAR methods). This reduction in the number of genes from the CEV-v1 library was the result of a combination of factors: 1) the use of more stringent statistical thresholds, 2) a fraction of genes that replicated in validation screens of the original cell lines but not in the additional cell lines 3) the exclusion of genes whose effects on cellular proliferation depended on *TP53* status. Together, these findings reveal a stringent set of species-specific genetic dependencies that emerged in recent human and chimpanzee evolution.

We first explored whether species-specific genetic dependencies could relate to changes in the coding sequence or regulation of the target genes themselves that might suggest divergent species-specific activities of these genes. Several genes in the set exhibited unexpected coding sequence changes. For example, *ASPM*, which causes microcephaly when mutated, contains protein domains with signatures of positive selection in the human lineage^60-63^ and was essential in human but not chimpanzee PSCs. Similarly, KATNA1, which physically interacts with ASPM to promote microtubule disassembly at mitotic spindle poles^64^, contains a nearly fixed modern human-specific mutation that is distinct from the Neanderthal and chimpanzee allele^65^ and acted as a suppressor of proliferation in chimpanzee but not human cells. However, these examples were exceptions, and signatures of adaptive selection, as well as overall non-synonymous substitutions, were depleted among the set of 75 species-specific genetic dependencies compared to the genome wide distribution (*P* < 0.01, *P* < 10^−6, respectively, Kolmogorov–Smirnov test, Figure S4A-B)^66^. Several genes with divergent genetic dependencies also displayed quantitative gene expression changes. For example, *MTCH2*, a gene involved in mitochondrial metabolism and apoptosis^67^ displayed significantly higher expression in human PSCs (fold change = 1.28, FDR < 10^−3^)^15^ and was specifically essential in human PSCs. In contrast, *ACAT2*, a gene involved in lipid metabolism, exhibited significantly higher expression in chimpanzee PSCs (fold change = 2.73, FDR < 10^−6^)^15^, but was also specifically essential in human cells. Despite these examples, the 75 gene set was also depleted for species differences in gene expression (*P* = 0.035, Figure S4C, two-tailed t-test). Together, these analyses suggest that coding or regulatory changes in CRISPRi target genes themselves do not account for the majority of species-specific growth differences we observed, and that a multitude of indirect effects driven by genetic background may underlie divergent dependencies^68^.

**Figure 3.**
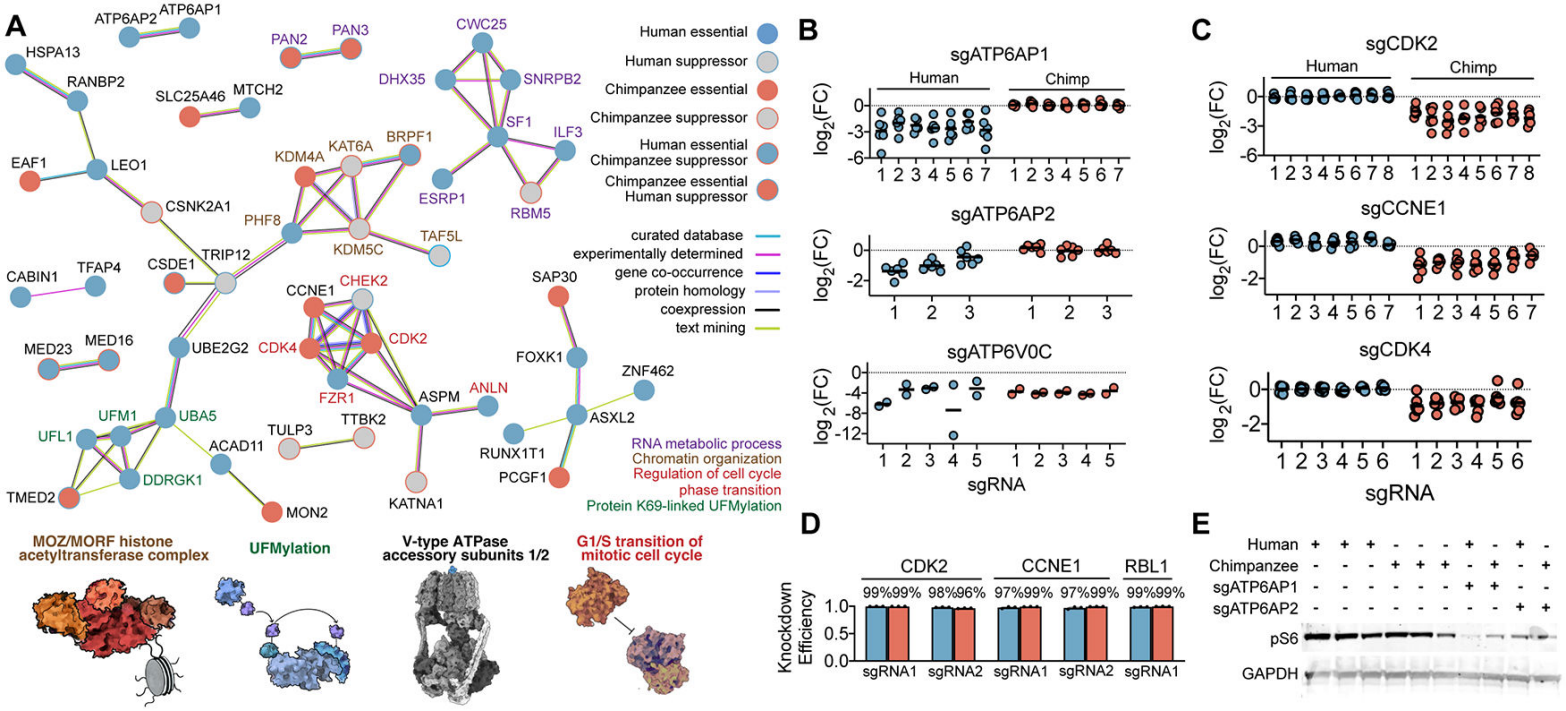
Core species-specific genetic dependencies (A) Species-specific genetic dependencies with STRING protein-protein associations. Illustrations of pathways and protein complexes with coherent species-specific effects. (B) Strip plots of log_2_ fold-change for sgRNAs targeting *ATP6AP1*, *ATP6AP2* and *ATP6V0C*. Data derived from CEV-v1 validation screens for *ATP6AP1*, *ATP6AP2* and only from primary genome-wide screen plotted for *ATP6V0C*. (C) Strip plots of log_2_ fold-change for sgRNAs targeting *CDK2*, *CCNE1*, and *CDK4*. Each circle represents the sgRNA log_2_ fold-change for one sgRNA in one human (blue) or chimpanzee (red) individual. Each stripplot contains a variable number of columns, corresponding to the number of significant sgRNAs targeting each gene. (D) qRT-PCR measurements of knockdown efficiency in human (28126B, blue, n = 3) and chimpanzee (40280L, red, n = 3) PSCs. (E) Western blot for phospho-S6 (pS6) expression and GAPDH loading control for three wild-type human (H1, 21792A, and 28126B) and three wild-type chimpanzee (3624K, 40280L, and 8861G) cell lines, and cell lines depleted for *ATP6AP1* or *ATP6AP2* (28126B and 40280L). See also Figures S4.

We next asked whether species-specific genetic dependencies involved groups of genes known to interact, a pattern that could suggest divergent requirements for conserved pathways. As essentiality phenotypes are typically shared among genes within known functional modules^69,70^, such coherence could also provide an additional test of internal consistency. Indeed, functionally related genes emerged with consistent patterns of depletion or enrichment within each species. Analysis using the STRING database^71^ revealed an enrichment for protein-protein interactions (*P* < 10^−6^, *P*-value calculated by string-db.org) and components of several well established biological processes (Figure 3A). For example, we observed that all five core components of the UFMylation pathway (*UFM1*, *UFL1, UFC1*, *UBA5*, *DDRGK1*) were essential only in human PSCs (Figure S4D). By contrast, all four subunits of the MOZ histone acetyltransferase complex (*KAT6A*, *BRPF1*, *ING5*, *EAF6*) acted as proliferation suppressors in chimpanzee PSCs (Figure S5D). Accessory proteins to the vacuolar-type ATPase (*ATP6AP1*, *ATP6AP2*) and the highest ranking DepMap co-dependent gene *WDR7*^36^, were specifically essential in human PSCs, whereas core subunits were essential in both species (Figure 3B). Strikingly, human PSCs were robust to depletion of cell cycle regulators cyclin-dependent kinase 2 (*CDK2*), its activating partner *Cyclin E1* (*CCNE1*), and cyclin-dependent kinase 4 (*CDK4*). For all three genes, we observed at least six sgRNAs that were essential across six chimpanzee individuals but nonessential across six human individuals (Figure 3C).

The consistent depletion of many sgRNAs targeting the same gene and multiple genes involved in the same biological process indicates that off-target activity is unlikely to explain proliferation differences between species. In principle, species-specific differences could also result from differential effectiveness of CRISPRi-mediated transcriptional repression (e.g., due to histone occupancy or transcriptional start site variability). To evaluate this possibility, we measured the efficacy of sgRNA-mediated repression for several candidate genes (*CDK2*, *CCNE1*, and *RBL1*). In all cases, measurements of transcript abundance by qRT-PCR revealed >90% knockdown in both species (Figure 3D, Table S3), suggesting that proliferation differences were not driven by incomplete knockdown efficiency in one species. In summary, these results highlight the ability of our screening approach to isolate biologically meaningful networks of genes that mediate species differences in cell behavior when perturbed, in contrast to gene expression profiling, which often reveals a complex mixture of both direct and indirect effects.

## Human-specific sensitivity to perturbations to lysosomal V-ATPase

While both our primary screen and validation screens measured growth and survival, changes in proliferation can reflect a wide range of cellular phenotypes, from differentiation to growth factor signaling. We next investigated the human-specific sensitivity to loss of *ATP6AP1* and *ATP6AP2*. ATP6AP1 and ATP6AP2 are accessory proteins to the lysosomal V-type ATPase. As the main proton pump responsible for maintaining the pH gradient of the lysosome, non-duplicated core subunits of the V-ATPase were essential in both species, as expected, (Figure 3B). Cryo-electron microscopy of the V-ATPase complex has implicated ATP6AP1 in the assembly of the V_0_ complex of the V-ATPase^72^. In addition, ATP6AP1 is comprised of a transmembrane helix and an extensive luminal domain that bears extensive structural homology to lysosomal-associated membrane proteins (LAMPs) and forms extensive contacts with ATP6AP2. Staining with LysoTracker Red and LysoSensor green in *ATP6AP1* depleted cells revealed no significant defects in maintenance of lysosomal pH (Figure S4E). These results are consistent with the core function of V-ATPase being strictly essential. However, loss of *ATP6AP1* has also been implicated in major cellular signaling pathways that are mediated by the lysosome^73-75^. Thus, we performed a western blot to measure phosphorylation of ribosomal protein RPS6 (pS6), a well-established downstream metric of mTORC1 activity. Depletion of *ATP6AP1* or *ATP6AP2* resulted in diminished pS6 signal in both species. However, pS6 was selectively abolished in human cells depleted for *ATP6AP1* (Figures 3E and S4F). These data thus link the human-specific growth defect of *ATP6AP1* sgRNAs observed in our pooled screens to an increased reliance of human cells on ATP6AP1-mediated mTORC1 signaling.

## Human PSCs are robust to depletion of CDK2 and Cyclin E

**Figure 4.**
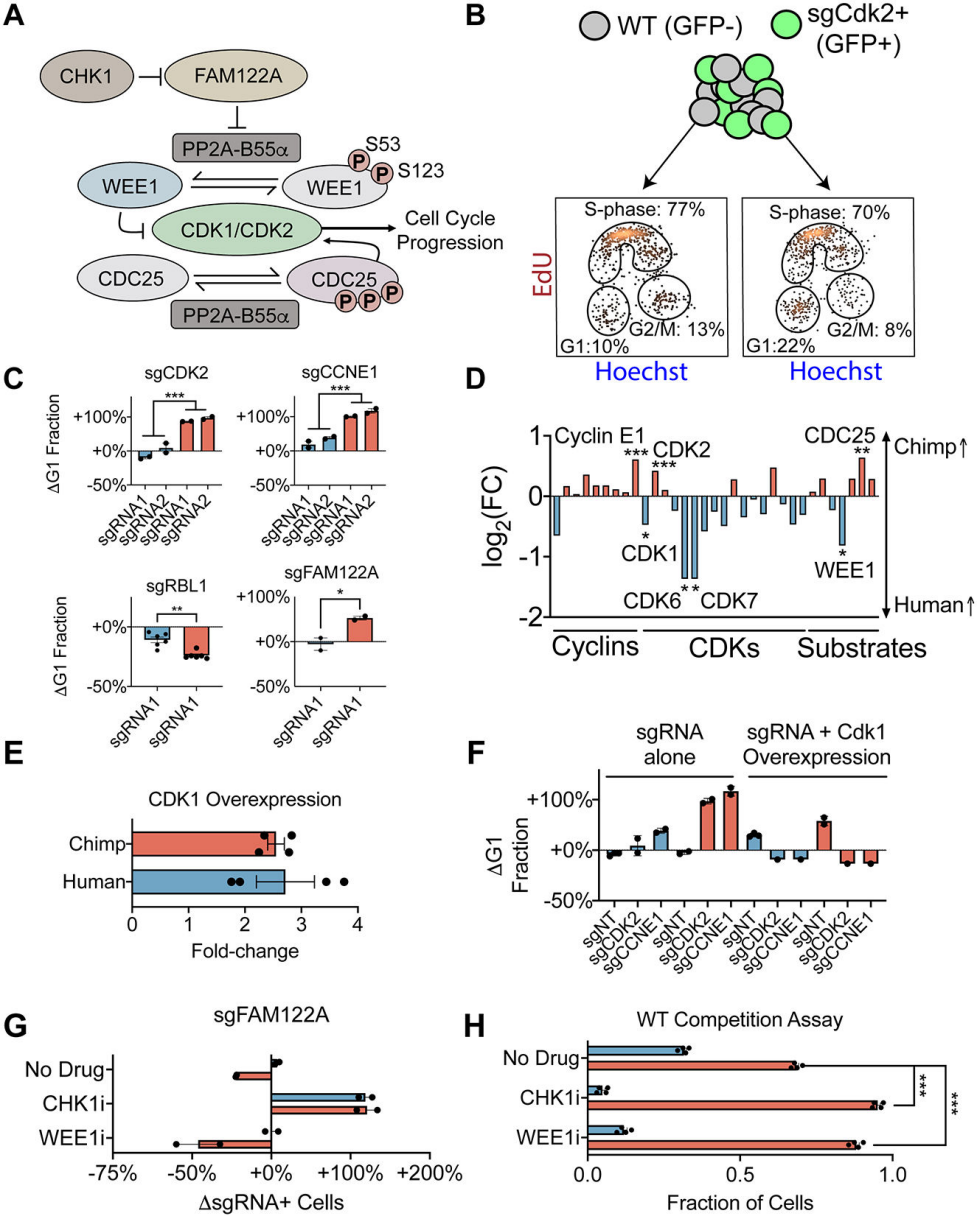
Divergent regulation of cell cycle progression in human and chimpanzee cells (A) Schematic for CDK1/CDK2 regulatory network. CDK1 and CDK2 phosphorylate key substrates WEE1 and CDC25, leading to degradation of WEE1 and activation of CDC25. Phosphatase PP2A dephosphorylates WEE1 and CDC25 at the same sites. CHK1 inhibits FAM122A, and FAM122A inhibits PP2A. (B) Cell cycle proportions in chimpanzee wild-type cells (GFP−) and sgRNA containing cells (GFP+) grown in co-culture. (C) Change in the fraction of human (28126B, blue) and chimpanzee (40280L, red) cells in G1 phase upon knockdown of *CDK2* (*P* < 10^−3^, n = 2), *Cyclin E1* (*P* < 10^−3^, n = 2), *RBL1* (*P* < 10^−2^, n = 6), and *FAM122A* (*P* < 0.05, n = 2), calculated by two-tailed t-test. (D) Comparative gene expression data from human and chimpanzee PSCs for core cell cycle regulators (* *P* < 0.05, ** *P* < 10^−2^, *** *P* < 10^−3^, *P*-values calculated in Gallego Romero et al., 2015). (E) qRT-PCR measurements of the degree of *CDK1* overexpression (n = 2 with two qRT-PCR primer sets, Table S3). (F) Change in the fraction of cells in G1 phase upon overexpression of *CDK1* in conjunction with *CDK2* or *Cyclin E1* knockdown. (G) Change in the fraction of FAM122A sgRNA containing cells in the presence of no drug, C1 inhibitor prexasertib (CHK1i), or WEE1 inhibitor adavosertib (WEE1i) (n = 2, two days of drug treatment). (H) Fraction of wild-type human (blue, 21792A) vs. wild-type chimpanzee (red, 40280L) cells grown in co-culture in the presence of no drug CHK1i, or WEE1i (n = 4). For all bar charts, data are plotted as mean ± s.e.m with individual data points representing biological replicates. See also Figure S5, and Video S1.

We next investigated whether chimpanzee-specific sensitivity to loss of several cell cycle genes could be linked to changes in cell cycle progression following repression of these genes (Figure 4A). To do so, we measured the proportion of cells in G1, S-phase and G2/M via incorporation of the thymidine analogue 5-ethynyl-2′-deoxyuridine (EdU) and Hoechst, a DNA-binding dye. Consistent with the early mammalian embryo, PSCs undergo rapid cell cycle progression with a shortened G1 phase compared to somatic cells^76^. For wild-type cells, only ~10% of cells were classified in G1 phase (Figure 4B). However, the absolute fraction of G1 cells was influenced by environmental factors such as confluence and nutrient availability (Figure S5A). Therefore, we measured the effect of CRISPRi-mediated gene repression in an internally controlled co-culture, with wild-type cells (GFP−) and sgRNA-expressing cells (GFP+) mixed within the same well. Knockdown of *CDK2* or *Cyclin E1* in chimpanzee PSCs led to a roughly two-fold accumulation of cells in G1 (Figure 4C; *P* < 10^−3^ for both, two-tailed t-test), consistent with the well-established role of Cyclin E1-CDK2 in regulating the G1/S transition. By contrast, knockdown of *CDK2* in human PSCs had no effect on cell cycle progression, and knockdown of *Cyclin E1* produced only a limited accumulation of G1 cells. We confirmed that these differences were not mediated by incomplete sgRNA-mediated knockdown in human PSCs (Figure 3D). In addition, differences in cell viability were minor and no species differences in G1 proportions were observed in the absence of cell cycle perturbations (Figures S5B and S5C). Lastly, we confirmed that the sgCDK2-mediated increase in G1 length could be observed in chimpanzee cells via live imaging with a FUCCI reporter (Figure S5D and S5E; Videos S1)^77^. Thus, our data suggest that human PSCs are less dependent on Cyclin E1-CDK2 for G1/S phase transition than chimpanzee PSCs.

Cyclin E1-CDK2 is a central regulator of the G1/S cell cycle transition^78^ and is commonly essential^35^ and frequently dysregulated across human cancer cell lines^79^. In contrast, *Cdk2*^−/−^ knockout mice are fully viable and develop normally, though with reduced body size^80,81^. Subsequent studies showed that cell cycle progression could be rescued in the absence of Cyclin E1-CDK2 by Cyclin A-CDK1 and Cyclin E-CDK1 activity^82,83^. Therefore, we reasoned that human cells might compensate for the loss of Cyclin E1-CDK2 via stronger Cyclin A2-CDK1 activity, as cyclin homologs Cyclin E2 and Cyclin A1 are not expressed in PSCs. Consistent with this model, *CDK1* was more highly expressed in human PSCs (FDR < 10^−2^), while *CDK2* and *Cyclin E1* were more highly expressed in chimpanzee PSCs (FDR < 10^−3^) (Figure 4D and Figure S5F). As a functional test, we overexpressed *CDK1* in chimpanzee PSCs in conjunction with sgRNA-mediated repression of *CDK2* or *Cyclin E1* and quantified the progression of cells through G1 phase. We found that 2.5-fold overexpression of *CDK1* was sufficient for rescuing the sensitivity of chimpanzee PSCs to *CDK2* or *Cyclin E1* depletion and accelerated progression through G1/S phase transition (Figures 4E and 4F).

Next, we extended our co-culture studies to additional cell cycle regulators with known interactions with Cyclin E-CDK2. Given the dependence of chimpanzee PSCs on cyclin E-CDK2, we reasoned that repression of an inhibitor of this Cyclin-CDK complex might have species-specific effects on cell cycle progression. We first investigated the consequences of repressing retinoblastoma-like 1 (*RBL1*/p107), a tumor suppressor homologous to retinoblastoma protein (*RB*). RBL1, like RB, represses cell cycle via inhibition of E2F transcription factors^84-86^. However, E2F is de-repressed in rapidly-dividing stem cells compared to other cell types due to the need for rapid cell cycling^87^. Indeed, repression of *RB* did not elicit a growth effect in either species (Figure S5G), consistent with E2F de-repression and prior studies of PSCs^88,89^. In contrast, RBL1 possesses an ability unique among RB family proteins to directly inhibit the kinase activities of cyclin A/E-CDK2^90^. Repression of *RBL1* resulted in faster growth and a reduction in the fraction of cells in G1 in both species (Figure 4C). However, *RBL1* effects were larger for chimpanzee cells (P < 0.01, two-tailed t-test). Given the accumulation of chimpanzee PSCs in G1 upon repression of *Cyclin E* or *CDK2* (Figure 4C), these results further support a model in which cyclin E-CDK2 exerts greater control over G1/S transition in chimpanzee compared to human PSCs.

In addition, we examined the chimpanzee-specific sensitivity to depletion of *FAM122A*, a cell cycle regulator that acts upstream of *CDK1/2* via interactions with *CHK1* and *PP2A* (Figure 4A). FAM122A acts as an inhibitor of phosphatase PP2A-B55α^91^, which in turn acts in opposition to CDK1 and CDK2 by dephosphorylating key substrates such as WEE1 and CDC25^92^ (Figure 4A). We observed that loss of *FAM122A* phenocopied loss of *CDK2* and led to accumulation of G1 cells in chimpanzee PSCs, but not in human (Figure 4C; P < 0.05, two-tailed t-test). This species difference appeared to be independent of WEE1 accumulation as *FAM122A*-depleted chimpanzee cells exhibited increased sensitivity to the WEE1 inhibitor adavosertib (Figure 4G)^93^, suggesting that the effects of *FAM122A* loss may depend on other PP2A targets, such as CDC25A. Moreover, we observed that *FAM122A* depletion in PSCs of both species promoted resistance to the CHK1 inhibitor prexasertib (Figure 4G)^94^, highlighting the conserved upstream interaction between FAM122A and CHK1.

Finally, we applied pharmacological approaches to examine species differences in enforcing cell cycle checkpoint. Because rapid cell divisions render PSCs sensitive to replication stress and DNA damage^95-98^, we tested whether wild-type human and chimpanzee PSCs would respond differently to CHK1 and WEE1 inhibition in the absence of genetic perturbations. To do so, we mixed GFP-tagged human cells with mCherry-tagged chimpanzee cells in a ~50/50 co-culture competition experiment. With no drug treatment, the proportion of human and chimpanzee cells remained unchanged. However, upon either prexasertib or adavosertib treatment, we observed substantially higher survival of chimpanzee cells compared to human cells (Figure 4H). Collectively, these genetic and pharmacological results suggest that chimpanzee PSCs may enforce a more robust S phase and G2/M checkpoint compared to human PSCs, with higher endogenous CDK1 levels providing a potential mechanism for human PSCs to overcome inactivation of *CDK2*, *FAM122A*, *CHK1*, or *WEE1* to promote cell cycle re-entry.

## Cell cycle perturbations alter neural progenitor cell expansion

**Figure 5.**
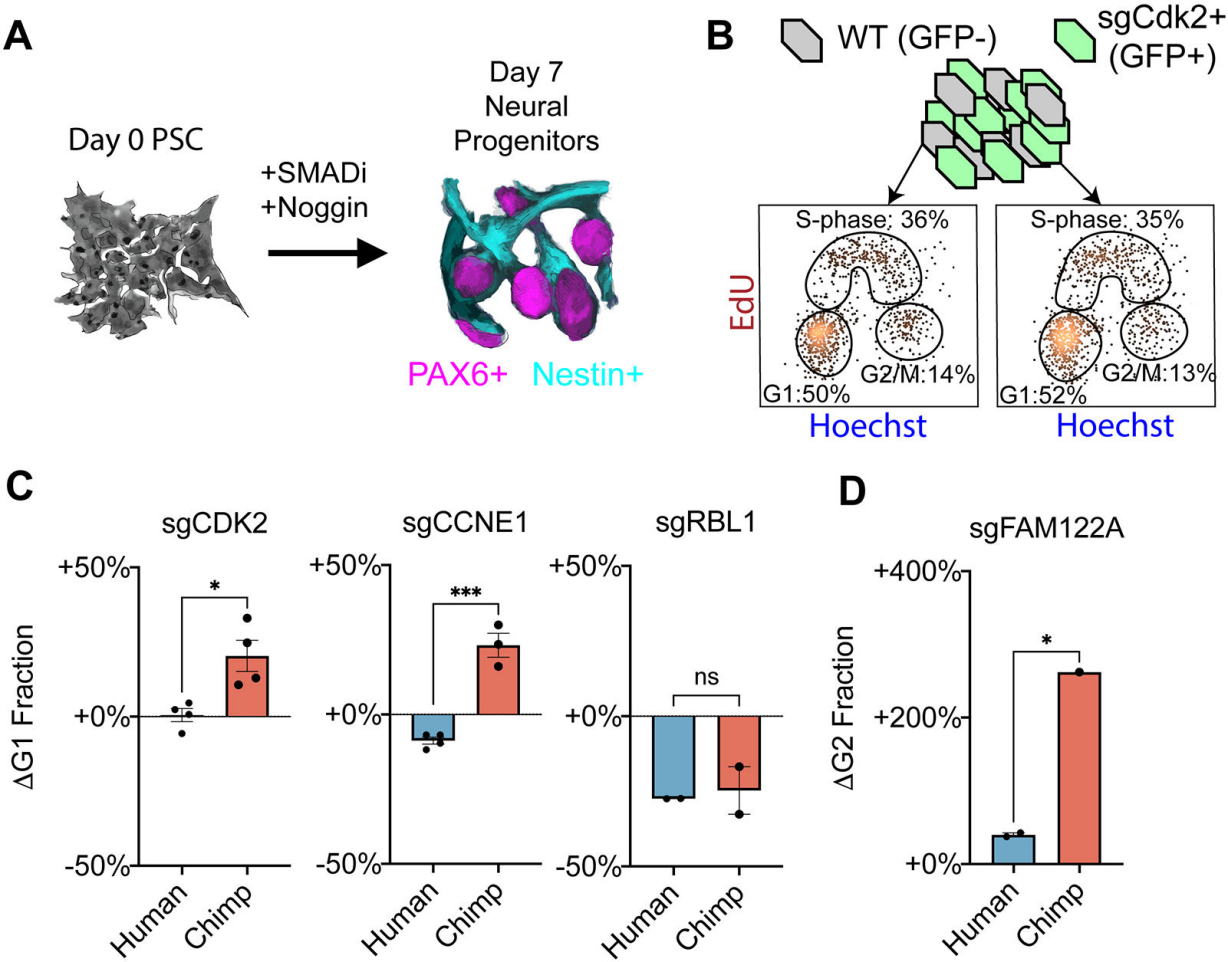
Human-specific robustness to cell cycle perturbations persist into neural progenitor cells (A) Schematic for differentiation of PSCs into neural progenitor cells (NPCs). (B) Cell cycle proportions in human wild-type neural progenitor cells (GFP−) and sgRNA containing cells (GFP+) grown in co-culture. (C) Change in the fraction of human (28126B, blue) and chimpanzee (40280L, red) NPCs in G1 phase upon depletion of *CDK2* (*P* < 0.05, N=4), *Cyclin E1* (*P* < 10^−3^, n = 4 and n = 3), or *RBL1* (n.s., n = 2), calculated by two-tailed t-test. (D) Change in the fraction of NPCs in G2 phase upon depletion of *FAM122A* (*P* < 0.05, two-tailed t-test, n = 2 and n = 1). Bar charts in (C) and (D) plotted as mean ± s.e.m, with individual data points representing biological replicates. See also Figure S6.

We wondered whether the molecular differences that we observed between species in stem cells would also manifest in differentiated cell types. As differences in G1/S regulation have long been hypothesized as an evolutionary mechanism for changing brain size^99-102^, we investigated whether human-specific robustness to depletion of cell cycle factors would persist in neural progenitor cells (NPCs). Previous studies have established both the necessity and sufficiency of genes promoting G1/S transition for proliferative NPC divisions in animal model systems^100-106^. However, it is not known whether human NPCs possess recently evolved characteristics that imbue them with an enhanced ability to maintain proliferative divisions. We generated CRISPRi human and chimpanzee NPCs and assessed how depletion of *Cyclin E1*, *CDK2*, *RBL1*, and *FAM122A* affected cell cycle progression and self-renewal (Figures 5A-D; Figures S6A-C). In contrast to PSCs, NPCs undergo substantially slower progression through cell cycle, with ~50% of cells in G1 phase compared to ~10% in PSC (Figure 5B). Nonetheless, knockdown of *Cyclin E1* (*P* < 0.05, two-tailed t-test) or *CDK2* (*P* < 10^−3^, two-tailed t-test) caused an additional accumulation of chimpanzee, but not human, NPCs in G1 (Figure 5C). Meanwhile, *RBL1* knockdown reduced the fraction of G1 cells in both human and chimpanzee (Figure 5C). Depletion of *FAM122A* resulted in G2/M accumulation in chimpanzee but not human NPCs (Figure 5D), implying a greater role for PP2A activation at G2 in chimpanzee NPCs compared to PSCs.

**Figure 6.**
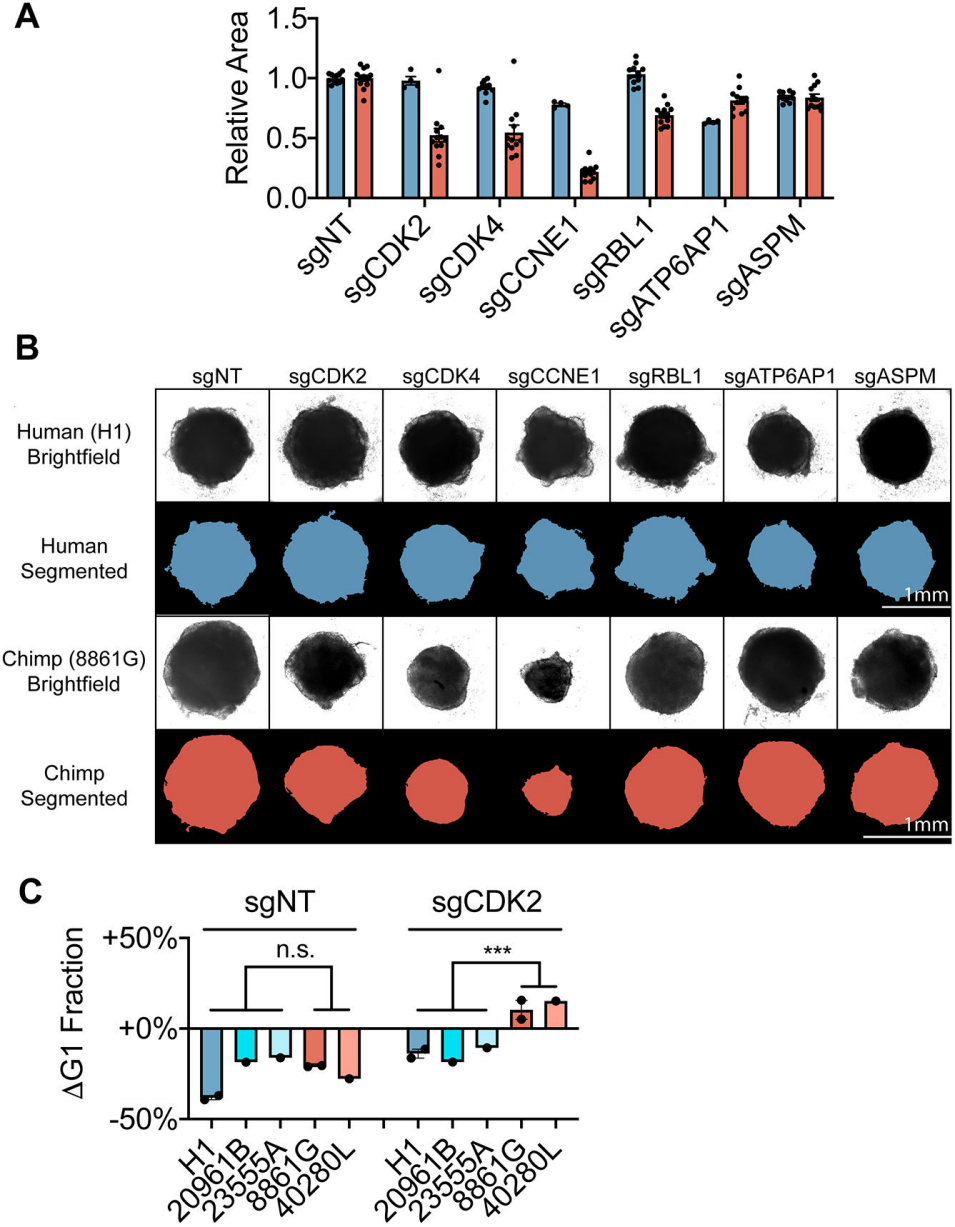
Human robustness to cell cycle perturbations in cerebral organoids (A) Organoid size measurements for human (H1) and chimpanzee (8861G) cerebral organoids, measured on day 18 by brightfield microscopy (n = 4 to 12). Bar charts plotted as mean ± s.e.m, with each individual data point representing an independent organoid. (B) Representative images of organoids from each sgRNA condition and corresponding image segmentation. (C) Cell cycle measurements for day 9 human (H1, 20961B, and 23555A; n = 2, n = 1, n = 1; blue) and chimpanzee (8861G and 40280L; n = 2 and n = 1; red) organoids. Change in the fraction of cells in G1 phase in cells expressing an sgRNA targeting *CDK2* (*P* < 0.001, two-tailed t-test) or a non-targeting sgRNA (*P* = 0.56, two-tailed t-test).

We further tested the species-specific responses to genetic perturbations in cerebral organoids. Quantifications of organoid size at day 18 revealed that human organoid development was robust to depletion of *CDK2*, *CDK4*, or *CCNE1* but sensitive to depletion of *ATP6AP1* (Figures 6A, 6B, and S9D). However, chimpanzee organoids seeded with cells expressing sgRNAs targeting *CDK2*, *CDK4*, or *CCNE1* were substantially smaller. To examine whether these size differences arose from changes in growth at the earliest stages of patterning versus ongoing differences in neural progenitor proliferation, we measured G1-phase length in day 9 organoids. We confirmed that depletion of *CDK2* in chimpanzee organoids continued to impair proliferation and resulted in a larger fraction of cells in G1 phase, with no such effect observed in the corresponding human organoids (Figure 6C). These results suggest that the increased robustness of human NPCs to depletion of regulators of G1/S progression could potentially bias human cells towards prolonged proliferative divisions, as has been proposed by developmental models.

## Evolutionary origin of molecular species-differences

**Figure 7.**
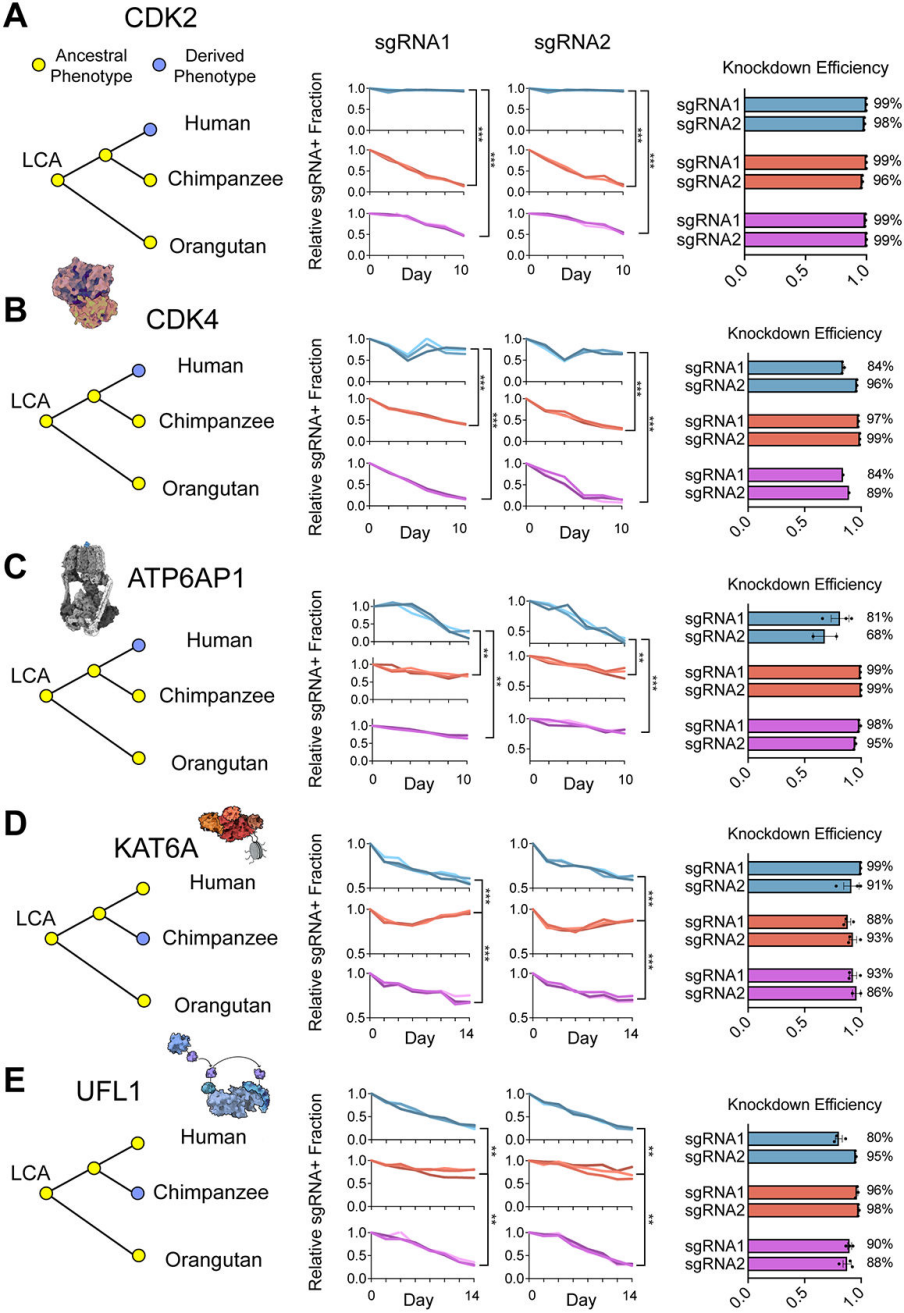
Orangutan PSCs suggest evolutionary origin of species-specific genetic dependencies (A) Change in the relative fraction of *CDK2* sgRNA containing cells over time in human (blue, 28126B), chimpanzee (red, 40280L), and orangutan PSCs (purple, n = 3 for each species) (** *P* < 10^−2^, *** *P* < 10^−3^, *P*-values calculated by two-tailed t-test on final timepoint). qRT-PCR measurements of sgRNA knockdown efficiency for each sgRNA in all three species (n = 1 to 3). (B-E) Relative sgRNA fraction over time and qRT-PCR measurements for sgRNAs targeting (B) *CDK4*, (C) *ATP6AP1*, (D) *KAT6A*, and (E) *UFL1*. Individual data points represent biological replicates and qRT-PCR bar charts plotted as mean ± s.e.m. See also Figure S7.

To determine the evolutionary origin of human- and chimpanzee-specific genetic dependencies, we extended our comparative studies to orangutan PSCs^107^. While humans and chimpanzees diverged roughly seven million years ago^108^, orangutans diverged from other great apes 13-18 million years ago^109^. Thus, we could infer by maximum parsimony that any genetic dependencies shared between orangutans and chimpanzees but not humans were likely to have been present in the common ancestor and subsequently diverged in the human lineage. We performed three-way species comparisons across genes representing several biological processes with coherent species differences in our dataset using sgRNAs with perfectly matched targets in all three species. For two sgRNAs targeting *CDK2*, we observed a significant depletion of sgRNA-expressing cells over the course of ten days in both chimpanzee and orangutan PSCs (Figure 7A and S7A). In contrast, no such depletion was observed in human PSCs. We further confirmed that the differences we observed were not due to differences in sgRNA activity, as knockdown efficiency exceeded 90% in all three species. In addition, we further observed human-specific robustness to repression of *CDK4* (Figure 7B) and *Cyclin E1* (Figures S7B and S7C). Based on these data, we inferred that robustness to perturbations of the G1/S transition evolved along the human lineage, otherwise dependence on *CDK2*, *CDK4*, and *Cyclin E1* would have had to evolve on two separate occasions in the chimpanzee and orangutan lineages with the same direction of effect for each gene. Next, we evaluated the human-specific sensitivity to repression of *ATP6AP1*. We observed that the *ATP6AP1* sensitivity was not shared by chimpanzee or orangutan PSCs, suggesting that altered responses to cellular metabolism, including the increased reliance on ATP6AP1 for mTORC1 signaling that we observed, also evolved along the human lineage (Figure 7C).

By contrast, repression of *KAT6A* promoted proliferation in chimpanzee PSCs but not in human or orangutan PSCs, arguing that this molecular feature was derived in chimpanzees (Figure 7D). Similarly, sensitivity to *UFL1* repression was common to human and orangutan PSCs but diverged in chimpanzee PSCs (Figure 7E). In sum, our data indicate that distinct genetic dependencies arose recently in both the human and chimpanzee lineages, highlighting the importance of experimentally defining the extent, identity, and phylogenetic origin of cellular and molecular differences derived in humans to inform our understanding of human evolution.

## Discussion

Loss-of-function screens have provided fundamental insights into the genes that regulate the development of model organisms. Here, we applied genetic screens to human and chimpanzee PSCs to examine whether the requirements for essential genes could differ in closely related species. By performing paired genome-wide CRISPRi screens, we uncovered a landscape of divergent genetic dependencies. Despite human and chimpanzee PSCs being similar in their cellular morphology, response to *in vitro* differentiation protocols, and core set of essential genes, we identified 75 genes with divergent roles in controlling cellular proliferation. We observed that many of these genes were organized in coherent protein complexes and biochemical pathways. By contrast, existing state-of-the-art comparative approaches, including RNA-seq and chromatin state profiling^20,22^ have identified thousands of differentially expressed and accessible genes between humans and nonhuman primates, but are unable to directly evaluate the role of each gene in key cellular processes such as survival, proliferation, and differentiation. In addition, limited coherence among differentially expressed genes makes it difficult to pinpoint divergent pathways or protein complexes. Our data thus comprise a rich resource that interfaces with existing studies of gene regulation and chromatin states and provide a functional genomics guide for future candidate gene approaches.

How might the genetic dependencies we observed in PSCs relate to organismal differences that manifest during development? Intriguingly, one of the strongest observations that emerged from our unbiased genome-wide screening approach was human-specific robustness to depletion of cell cycle factors, which persisted in neural progenitor cells. This finding aligns with long-standing hypotheses that changes in cell cycle regulation could play a role in human-specific cortical expansion. The G1-phase length hypothesis proposes that factors which lengthen G1 duration in NPCs increase the probability of differentiation towards non-proliferative neuronal fates, while factors reducing G1 length promote proliferative self-renewal of NPCs^99,102,103^. Indeed, loss of *CDK2* or *CDK4* in mouse NPCs prolongs G1 length and causes premature neuronal differentiation at the expense of self-renewal^105^. Conversely, exogenous overexpression of *CDK4* and *Cyclin D1* in mouse and ferret reduces G1 length, promotes self-renewing divisions in basal progenitor cells, and results in increased brain size and cortical area, while preserving a structurally normal, six-layered cortex^106^. In humans, mutations that promote cyclin D2 stability lead to megalencephaly^110^. These studies underscore the influence of inputs to the G1/S transition on brain expansion during development^101^. However, whether this developmental mechanism changed specifically in recent human evolution remained unexplored. Our demonstration that human NPCs are more likely than chimpanzee NPCs to continue cycling upon equivalent repression of *CDK2* or *Cyclin E1* connects proposed developmental mechanisms to molecular changes that occurred in human evolution. Although physiological stressors do occur during development that influence the size of the neural progenitor pool^21,111-113^, it remains unknown how external environmental stimuli or the intrinsic tempo of differentiation might differ between humans and chimpanzees and interface with the changes in G1 regulation that we observed. Human-specific genetic dependencies could result from evolutionary changes in cell behavior or developmental systems drift, a process in which cell behaviors are conserved, but the underlying circuitry changes^114,115^. Regardless of the impact on cell behavior, the altered genetic dependencies or drug sensitivities represent recently evolved substrates for disease vulnerabilities and further evolutionary changes^116^. We expect future studies connecting the response of human and chimpanzee NPCs to a wider range of genetic and physiological perturbations will provide further insights into the evolutionary mechanisms by which the proliferative capacity of NPCs has increased along the human lineage.

The endeavor to study the molecular basis of human evolution has been compared to searching for needles in a haystack, as human-specific genetic variants and gene expression changes are numerous and predominantly neutral^2^. By contrast, our finding that human and chimpanzee PSCs exhibit distinct genetic dependencies, even for genes that lack clear expression or protein-coding sequence divergence, provides a complementary approach for isolating recently evolved functional changes in human gene networks. This conceptual advance mirrors the progression of cancer genetics research from sequencing and transcriptomics efforts such as TCGA^117^ to functional genetics-based efforts such as DepMap^35,36^. Moreover, while driver mutations can be identified in tumors based on their independent recurrence, human evolution has occurred only once, highlighting the added value of a functional genomics platform. We expect that loss-of-function profiling can be extended to cellular models of genetic variation and disease risk within humans to identify shared vulnerabilities and convergent pathway level differences. Lastly, our approach can be readily applied in differentiated cell types and interfaced with higher dimensional measurements of cell phenotypes^118-120^, opening the door to future efforts for understanding molecular control of species differences across stages of development.

## Limitations of the Study:

While our study systematically maps species-specific genetic dependencies in pluripotent stem cells, it is unclear which dependencies will persist across diverse differentiation trajectories and whether new cell type-specific dependencies will arise. We applied a principled and systematic exploration of the changes in the genetic landscape that evolved during the short divergence time between humans and chimpanzees. However, to further connect human-specific robustness to depletion of cell cycle regulators to physiological differences in brain development, we will need to understand how naturally occurring environmental factors can produce species differences in NPC cell cycle properties and proliferative potential. In addition, the *in vitro* models of NPCs that we employed produce homologous cell types to those found *in vivo*, but may exhibit differences in metabolism, spatial architecture, and maturation speed. Finally, our primary screen unexpectedly included two P53-non-responsive chimpanzee cell lines, which we accounted for by constructing four additional P53-responsive cell lines in the validation screen. The frequency of pro-survival adaptations in PSCs highlights the need for multiple representatives of each species and quality controls (such as the Nutlin-3a assay) to distinguish species differences from line-to-line variation.

## STAR METHODS

### RESOURCE AVAILABILITY

#### Lead Contact

Further information and requests for resources and reagents should be directed to and will be fulfilled by the Lead Contact, Alex A Pollen (Alex.Pollen@ucsf.edu).

#### Materials availability

Materials used in this study will be provided upon request and available upon publication.

#### Data and code availability

- Raw sequencing data are deposited on GEO accession number GSE212297.
- All code for the analyses performed on the CRISPRi screens is publicly available at https://github.com/tdfair.
- Any additional information required to reanalyze the data reported in this paper is available from the lead contact upon request.

### EXPERIMENTAL MODEL AND STUDY PARTICIPANT DETAILS

#### Cell Lines

Human embryonic cell line H1 (WiCell), human induced pluripotent stem cell lines (20961B, 21792A, 23555A, 28126B, WTC11), and chimpanzee induced pluripotent stem cell lines (3624K, C3649, 40280L, 40290F, 8861G, Pt5-C).

Additional details for cell lines used in this study are provided in Supplementary Table S1.

#### Media Formulations

mTESR1 was purchased from Stem Cell Technologies (cat. 85850) and supplemented with 100 units/ml penicillin, 100 μg/ml streptomycin, and 292 μg/ml L-glutamine (Gibco, cat. 10378016). StemFlex was purchased from Gibco (cat. A3349401) and supplemented with 100 units/ml penicillin, 100 μg/ml streptomycin, and 292 μg/ml L-glutamine. HEK293Ts were cultured in DMEM (ThermoFisher, cat. 11965118) and supplemented with 10% FBS (VWR, cat. 97068-085, lot 043K20), 100 units/ml penicillin, 100 μg/ml streptomycin, and 292 μg/ml L-glutamine. Neuronal differentiation media was prepared as described in^129^, with DMEM F/12 (ThermoFisher, cat. 21331020), CTS Neurobasal Medium (ThermoFisher, cat. A1371201), 1x N-2 supplement CTS (ThermoFisher, cat. A1370701), 10 μM SB431542 (StemMACS, TGFβ inhibitor; Miltenyi, cat. 130-106-543), 100 ng/ml Noggin (recombinant human; Miltenyi, cat. 130-103-456), and 100 units/ml penicillin, 100 μg/ml streptomycin, and 292 μg/ml L-glutamine. Thiazovivin (Stem Cell Technologies, cat. 72252) was included at a concentration of 2 μM during passaging. The CEPT cocktail^130^ consisting of 50 nM chroman 1 (Tocris Bioscience, cat. 7163), 5 μM emricasan (Selleck Chemicals, cat. S7775), polyamine supplement diluted 1:1000 (Sigma-Aldrich, cat. P8483), and 0.7 μM trans-ISRIB (Tocris Bioscience, cat. 5284) was included during single cell sorting and lipofection.

Cerebral organoids were seeded in Sasai media #1 (GMEM supplemented with 20% KSR, 1x Penicillin/Streptomycin/Glutamate, 0.11 mg/mL sodium pyruvate, 0.1 mM β-mercaptoethanol, 10 μM thiazovivin, 5 μM SB431542, 200 nM LDN-193189, and 1 μM Wnt-C59)^131^. Wnt inhibitor Wnt-C59 was withdrawn after day 6.

#### Construction of CRISPRi cell lines

All wildtype cell lines tested negative for mycoplasma prior to the start of cell line engineering. The CRISPRi effector protein dCas9-KRAB (*KOX1*) was introduced into either the *CLYBL* or *AAVS1* safe harbor locus^132-135^. For the AAVS1 locus, cell lines were constructed via lipofection of three plasmids: 1) A modified version of pX458 (Addgene #48138), containing both Cas9 nuclease and a sgRNA targeting AAVS1. The sgRNA spacer was modified from the original plasmid by cutting with type IIS restriction endonuclease BbsI-HF. Complementary oligos containing the sgRNA spacer and proper overhangs were annealed and ligated with T4 ligase. 2) A modified version of the previously published Gen3-AAVS1 vector^122^ containing an optimized dCas9-XTEN-KRAB-P2A-EGFP or mCherry^124^ driven by the chicken beta-actin (CAG) promoter, flanked by homology arms to AAVS1. 3) pEF1-BCL-XL, a plasmid expressing BCL-XL, the anti-apoptotic isoform of BCL2L1, from the EF-1α promoter. For the *CLYBL* locus, four plasmids were lipofected based on previously published methods^38^: 1) pZT-C13-L1 (Addgene #62196) 2) pZT-C13-R1 (Addgene # 62197) , with plasmids 1 and 2 encoding TALENs that target the *CLYBL* locus. 3) dCas9-XTEN-KRAB-P2A-BFP (Addgene #127968) and 4) pEF-BCL-X. Lipofection was performed as follows: two days prior to transfection, cells were switched into mTESR1 media on a non-passaging day. One day prior to transfection, ~400,000 cells were plated into a Matrigel-coated (Corning, cat. 354230) 6-well plate with mTESR1 supplemented with 2 μM thiazovivin. On the day of transfection, a 3 μg mixture of plasmids 1-3 was made at a mass ratio of 5:5:1, added to a mixture of 96 μl Opti-MEM (Gibco, cat. 31985062) and 4 μl Lipofectamine Stem (ThermoFisher, cat. STEM00003), and incubated at room temperature for 10 minutes. Media was aspirated from the PSC plate and replaced with 2 ml Opti-MEM supplemented with CEPT. The lipid/plasmid DNA complexes were then added to plate and incubated for 4 hours, after which 2 ml of mTESR1 supplemented with CEPT was overlaid. 24 hours post-transfection, media was replaced with StemFlex supplemented with CEPT. 48 hours post-transfection, media was replaced with StemFlex supplemented with 2 μM thiazovivin, and cells were then passaged for 10-14 days to dilute out the transfected plasmids. Single-cell clones and one polyclonal population per cell line were then sorted on a Sony MA900 (see Table S1). Expanded populations were cryopreserved in Bambanker preservation media (ThermoFisher, cat. 50999554). Six CRISPRi engineered PSC lines (3624K, 8861G, 21792A, 40280L, 40290F and Pt5-C) were functionally validated with an sgRNA targeting B2M, a gene encoding a non-essential cell surface protein. B2M levels were measured by staining with an APC anti-human β2-microglobulin antibody (BioLegend, cat. 316312).

### METHOD DETAILS

#### Primers

All primers used in this study are listed in Supplementary Table 6.

#### Nutlin-3a pharmacological assay for testing TP53/MDM2 responsiveness

CRISPRi engineered cell lines were tested for TP53/MDM2 responsiveness based on sensitivity to Nutlin-3a, an active enantiomer of Nutlin-3, which is a small molecule MDM2 inhibitor that induces p53-dependent autophagy and apoptosis^54,55^. Cells were passaged with Versene (PBS-EDTA) to avoid use of ROCK inhibitor, which promotes survival. 80-100% confluent wells were split 1 to 1 and plated densely into new wells with StemFlex supplemented with 10 μM Nutlin-3a. Consistent with previous reports, TP53/MDM2 responsive cell lines underwent apoptosis within 24 hours.

#### Lentivirus production, concentration, and titration

Lentivirus for CRISPRi screening was produced in HEK293T cells. HEK293Ts were seeded at a density of 80,000 cells/cm^2^ 24 hr. prior to transfection in 15 cm dishes. Next, each dish was transduced with 20 μg sgRNA library, 6.75 μg standard lentivirus packaging vectors, and 81 μl Mirus transfection reagent (VWR, cat. 10767-122) in Opti-MEM. 24 hr. post-transfection, media was replaced and supplemented with 1X ViralBoost (Alstem, cat. VB100). Supernatant was collected at 48 hr. post-transfection and concentrated 1:10 with Lenti-X Concentrator (Takara Bio, cat. 631231). Concentrated lentivirus was titered in PSCs based on BFP expression 3 days post-infection using a flow cytometer.

#### Pooled genome-wide CRISPRi screening

CRISPRi PSCs expressing dCas9-KRAB were dissociated with Accutase (Innovative Cell Technologies, cat. AT104-500), resuspended in StemFlex supplemented with 2 μM thiazovivin and 5 μg/ml polybrene (Sigma-Aldrich, cat. TR-1003-G), transduced with the lentiviral hCRISPRi-v2 sgRNA library at a target infection rate of 25-40%, and plated in Matrigel-coated 5-layer cell culture flasks (Corning, cat. 353144) at a density of 65,000-80,000 cells/cm^2^. The following day, StemFlex medium was replaced. Two days after infection, cells were dissociated with Accutase, resuspended in StemFlex supplemented with 2 μM thiazovivin and 1.5 μg/ml puromycin (Goldbio, cat. P-600-100), and plated in 5-layer cell culture flasks. The following day, medium was replaced with StemFlex supplemented with 1.5 μg/ml puromycin. Four days after infection, 100 M cells were harvested for the initial time point (t_0_), while 250-300 M cells were resuspended in StemFlex supplemented with 1.5 μg/ml puromycin and plated in 5-layer cell culture flasks (>1000x sgRNA library representation). Selection efficiency was assessed by flow cytometry (>70% BFP+). Every two days, cells were dissociated with Accutase, resuspended in StemFlex supplemented with 2 μM thiazovivin, and plated at a density of 80,000-100,000 cells/cm^2^. Technical replicates were cultured separately for the duration of the screen. After 10 days of growth, 150 M cells from each technical replicate were harvested for the final time point (t_final_). Genomic DNA was isolated from frozen cell pellets using the Macherey-Nagel NucleoSpin Blood XL kit (Macherey-Nagel). Isolated DNA was quantified using a NanoDrop (ThermoFisher) and the sgRNA expression cassette was amplified by 22 cycles of PCR using NEBNext Ultra II Q5 Master Mix (NEB) and primers containing Illumina P5/P7 termini and sample-specific TruSeq indices. Each sample was distributed into 150-200 individual 100 μl reactions in 96-well plates, each with 10 μg genomic DNA as input. Following amplification, reactions from each sample were pooled and a 100 μl aliquot was purified using AMPure XP beads (Beckman-Coulter) with a two-sided size selection. Purified libraries were quantified by Qubit (ThermoFisher) and sequenced on an Illumina HiSeq 4000 instrument (SE50, 5% PhiX) with a custom sequencing primer (oCRISPRi_seq V5).

#### CEV-v1 validation screening library design

To validate genome-wide screens, a Comparative Essential Validation (CEV-v1) sgRNA library consisting of 9,692 sgRNAs targeting 963 candidate species-specific essential or proliferation suppressor genes was constructed. hCRISPRi-v2 sgRNAs with perfect-match targets in panTro6 exhibiting significant depletion or enrichment in the genome-wide screens were retained in CEV-v1 (*n* = 3589 sgRNAs). In addition, new sgRNAs with perfect-match target sites in the human (hg38) and chimpanzee (panTro6) reference genomes were chosen based on their position relative to the FANTOM-annotated transcriptional start site ^44^ on-target activity predicted by DeepHF^136^, and off-target potential predicted by a genome-wide search of mismatched target sites^58,137^ in both reference genomes. Briefly, after performing off-target filtering (one perfect-match target, CRISPRi specificity score > 0.20, maximal predicted activity at any off-target site < 0.80), candidate sgRNAs were categorized by their position relative to the FANTOM TSS and then ranked by their DeepHF score. A threshold DeepHF score was imposed by excluding sgRNAs with predicted activities less than one standard deviation below the mean of all candidate sgRNAs (minimum score: 0.4378). Eight sgRNAs were selected for each gene as well as 1845 non-targeting sgRNAs from hCRISPRi-v2. Oligonucleotide pools were designed with flanking PCR and restriction sites (BstXI, BlpI), synthesized by Agilent Technologies, and cloned into the sgRNA expression vector pCRISPRia-v2 (Addgene #84832) as described previously^32^.

#### Pooled validation CRISPRi screening

Validation screens were performed in conditions consistent with the genome-wide screens. Briefly, CRISPRi PSCs expressing dCas9-KRAB were dissociated with Accutase, resuspended in StemFlex supplemented with 2 μM thiazovivin and 5 μg/ml polybrene, transduced with the lentiviral CEV-v1 sgRNA library at a target infection rate of 25-40%, and plated in Matrigel-coated 3-layer cell culture flasks (Corning, cat. 353143) at a density of 65,000-80,000 cells/cm^2^. Cells were dissociated, plated, selected with puromycin, and grown on the same schedule as used for the genome-wide screens. Technical replicates were cultured separately for the duration of the screen and >1000x sgRNA library representation was maintained.

In addition to assessing the reproducibility of species-specific genetic dependencies across biological replicates (*n* = 5 human cell lines and *n* = 6 chimpanzee cell lines), we also assessed reproducibility between screens and across sites. 3 out of 4 PSC lines from the initial screen were re-tested with the CEV-v1 validation library. Several individual cell lines were screened twice (H20961B, H23555A, H28126B, C3624K, C8861G), with replicate screens were performed independently at the Whitehead Institute and UCSF (Table S1). 4 out of the 5 lines retested at both UCSF and Whitehead were independently constructed CRISPRi cell lines from the same source iPSC line, with CRISPRi machinery inserted at either the *AAVS1* or *CLYBL* locus with either GFP, mCherry, or BFP as fluorescent markers.

#### Quantitative RT-PCR

Triplicates of human (28126B), chimpanzee (40280L), and orangutan (11045-4593) PSCs were grown in a 6-well plate and infected with sgRNAs (see Table S3) at an MOI of ~0.3. 48 hours post-infection, cells were expanded in 2 μg/ml puromycin and allowed to recover for 48 hours. At post-infection day 4, sgRNA-expressing cells were sorted based on BFP+ expression using a Sony MA900. sgRNA-expressing cells were isolated by FACS as the depletion of cells containing essential sgRNAs occurred more rapidly than the removal of sgRNA-negative cells through puromycin selection.

Cells were then allowed to recover for 48-96 hours, until they reached ~60-80% confluence on a 6-well plate and were harvested 6 to 8 days post-infection. For each biological replicate, RNA was extracted with a Direct-zol RNA miniprep kit (Zymo Research, cat. R2051). RNA was reverse transcribed with SuperScript IV VILO (ThermoFisher, cat. 11756050), and cDNA was amplified with the DyNAmo ColorFlash SYBR Green kit (ThermoFisher, cat. F416L). Primers for GAPDH were used as loading controls and no-RT controls were performed to control for genomic DNA contamination. Amplifications were performed in duplicate and quantified on a QuantStudio Flex 7 Real-Time PCR system in 96-well plates. Final data points reported are averages of the two duplicate qRT-PCR amplifications.

While Figure 3D includes triplicate infections, the qRT-PCR experiments shown in Figure 7 contain four samples for which triplicates were not recovered. We report two replicates for *KAT6A* sgRNA2 orangutan PSCs and *ATP6AP1* sgRNA2 human (28126B) and only one measurement for *CDK4* sgRNA1 orangutan and *CDK4* sgRNA2 orangutan. The remaining conditions were performed in triplicate.

#### Copy number variation analysis

Chromosomal copy number variations (CNV) were inferred with the InferCNV R package (version 1.2.1), which predicts CNVs based on gene expression data. InferCNV was run in ‘subclusters’ analysis mode using ‘random_trees’ as the subclustering method with gene expression quantified for both species by alignment to the hg38 reference genome. Average gene expression across all six individuals in each species was used as the background column. The cut-off for the minimum average read count per gene among reference cells was set to 1, per software recommendation for bulk RNA-seq data. CNV prediction was performed with the ‘i6’ Hidden Markov Model, whose output CNV states were filtered with the included Bayesian mixture model with a threshold of 0.1 to find the most confident CNVs. All other options were set to their default values.

To check for copy number variation at a selected set of cell cycle-related genes, we analyzed whole-genome shotgun sequencing data. Genomic DNA from all libraries sequenced on an Illumina sequencer in 151 bp paired-end mode was provided for analysis courtesy of the laboratory of Gregory Wray and mapped to chimpanzee reference panTro6^138^ using bwa-mem2 (https://ieeexplore.ieee.org/document/8820962) with default parameters. PicardTools (https://broadinstitute.github.io/picard) was used to add read group information and mark duplicates, and baseline coverage histograms were generated using BEDTools genomecov^139^, from which the 5th, 50th, and 95th percentile of coverage for each library, both genome-wide and across chromosome X, were extracted. Gene-level features for all genes listed as cyclins, cyclin dependent kinases, and class III Cys-based CDC25 phosphatases in the HGNC database^140^ were selected from a recent chimpanzee gene annotation^141^ and the coverage at each base across the full length of each gene in the set for each library was counted and summed using samtools mpileup^142^. For this step, only primary alignments containing mapped reads not marked as duplicates, with minimum map quality of 20, were considered (samtools view -F1284 -q20).

#### RNA-seq library prep

Wild-type human and chimpanzee cells were growth to 70% confluence and RNA was extracted by adding RNAse-free Trizol (ThermoFisher, cat. 15596026) to each pellet and processing with the Zymo Research Direct-zol RNA miniprep kit (Zymo Research, cat. R2050). RNA-seq was performed using the Illumina TruSeq Stranded Total RNA kit (Illumina, cat. 20020599) according to the manufacturer’s instructions, with the exception of the final PCR step for which only 10 cycles were used to prevent overamplification. The final pooled library was sequenced on an Illumina HiSeq 2500 (SE50).

#### Western Blot

Human (28126B) and chimpanzee (40280L) PSCs were transduced with lentiviral constructs containing sgRNAs targeting *ATP6AP1* or *ATP6AP2*. One additional human (21792A) and chimpanzee (8861G) was also separately infected with an sgRNA targeting *ATP6AP1*. 48 hours post-infection, sgRNA-expressing cells were selected using 1.5 μg/ml puromycin. Cells were then recovered in normal growth media from 4 to 6 days post-infection. Cells were harvested at 6 days post-infection, along with separate wells of three wild-type human (H1, 21792A, 28126B) and three wild-type chimpanzee (3624K, 40280L, 8861G) cell lines. Day 6 was selected as a time point for harvesting because it is roughly the earliest time point at which a pure population of sgRNA expressing cells can be produced for western blots. In addition, growth for additional days can lead to larger differences in cell viability between species. Cells from each well of a 6-well plate were lysed in ~250 μl ice-cold RIPA buffer supplemented with protease inhibitor (ThermoFisher, cat. A32965). After 30 minutes of incubation in lysis buffer at 4°C, cells were centrifuged at 16,000 x g for 5 minutes at 4°C. Supernatant was collected and snap frozen in liquid nitrogen and stored at −80°C.

Protein concentrations in each lysate were quantified using a Bradford BCA kit (ThermoFisher, cat. PI23227) Lysate was normalized to 1 μg/μl in RIPA buffer. 30 μl of lysate was added to 10 μl of NuPage Sample Buffer (4x), heated to 70°C on a PCR thermocycler, and loaded onto a Bolt 4-12% polyacrylamide gel (ThermoFisher, NW04122BOX). The gel was run for 45 minutes at 165 V in MOPS buffer. Protein was then transferred onto a nitrocellulose membrane (BioRad, cat. 1704270) with a Bio-Rad Trans-Blot Turbo (BioRad, cat. 1704150). The membrane was blocked with Intercept (PBS) Blocking Buffer (LI-COR, cat. 927-90003) for 1 hour at RT. Membrane was incubated overnight at 4°C with anti-pS6 primary antibody at a 1:1,000 dilution. Membrane was washed 3x with TBST and incubated with secondary antibody at 1:15,000 dilution. Membrane was washed 3x with TBST and imaged on a LI-COR Odyssey CLX. Afterwards, antibodies were stripped from the membrane with NewBlot IR Stripping Buffer (LICOR, cat. 928-40028). Membrane was reblotted with anti-GAPDH antibody at 1:1,000 dilution and incubated overnight at 4°C. Membrane was washed 3x with TBST and incubated with secondary antibody at 1:15,000 dilution. Membrane was washed 3x with TBST and imaged on a LI-COR Odyssey CLX.

#### Cell death and viability analysis

Cell viability and cell death assays were performed on chimpanzee PSC line 40280L. Cells were infected with sgRNAs targeting cell cycle genes *CDK2*, *CDK4*, *CCNE1*, *RBL1*, or a non-targeting control sgRNA. Cells were infected in triplicate with sgRNAs at 0.3 MOI and selected with addition of 2 μg/ml puromycin from 2 to 4 days post-infection. Cells were recovered from day 4 to day 6 post-infection in StemFlex supplemented with 2 μM thiazovivin to match conditions from the growth screens. Cell viability was measured on a Countess 3 automated cell counter and a Chemometec Nucleocounter NC-202.

#### Cell cycle EdU staining

Human (28126B) and chimpanzee (40280L) PSCs were infected with pCRISPRia-v2 (Addgene Cat# 84832) containing: 1) a targeting sgRNA or 2) a non-targeting sgRNA or 3) a combination of an sgRNA and a *CDK1* overexpression ORF expressed from the EF-1α promoter typically used to express GFP and the puromycin resistance cassette. The *CDK1* ORF was expressed downstream of sfGFP-P2A and replaced the puromycin resistance cassette. The BFP marker from the original pCRISPRia-v2 construct was replaced with sfGFP to enable use of the BFP channel for Hoechst staining during the cell cycle assay. On the day of infection, cells were transduced with lentivirus and 8 μg/ml polybrene. Lentivirus was titered to MOI ~ 1, such that GFP+ sgRNA expressing cells represented between 25% and 40% of the population at 48 hours post-infection. No puromycin selection was performed to maintain a mixed co-culture of sgRNA expressing cells and uninfected cells. Cells were grown in StemFlex media supplemented with 2 μM thiazovivin on the day of infection and withdrawn via replacement of fresh media 24 hours post-infection. Cells were then assayed at Day 6, after being freshly split at Day 5 into ROCKi containing media to prevent over-confluence and nutrient depleted media. This time point was chosen because it is one of the earliest time points at which cells have recovered from lentivirus infection and after sgRNA-mediated gene repression has been fully activated. In addition, growth for additional days could lead to larger differences in cell viability or growth rate between species.

Cell cycle phase measurements were performed with the Click-iT Plus EdU Alexa Fluor 647 Flow Cytometry Assay Kit (ThermoFisher, cat. C10635). 10 μM EdU was added directly to each well without a fresh media change and cells were incubated for roughly 1 hour, after which cells were harvested with Accutase. The cell pellet was washed once with 500 μl PBS supplemented with 1% BSA, pelleted again, and fixed with 4% paraformaldehyde for 15 minutes at RT, protected from light. Cells were washed and permeabilized for 15 minutes at RT. EdU detection was then accomplished via click chemistry of an Alexa Fluor 647 to an EdU antibody, which was incubated with cells for 30 minutes at RT. After 1 wash, 10 μg/ml of Hoechst 33342 (ThermoFisher, cat. H3570) was added and incubated for 15 minutes. Cells were then directly analyzed by flow cytometry. Data were analyzed with custom MATLAB scripts – after filtering for viable cells and doublets, G1, S, and G2/M gates were manually drawn and saved with the function impoly() for each sample. sgRNA+ populations were determined by GFP+ expression, and identical G1, S, and G2/M gates were used for sgRNA+ cells and sgRNA− cells within each sample.

#### Cell cycle drug treatments

For Fig. 4H, human iPSC line H28126B was used and chimpanzee iPSC line C40280L was used. Cells were infected with an sgRNA targeting *FAM122A* (see Table S3), with 15-30% of cells infected. No puromycin selection was performed. At day 4 post-infection, cells were Accutase passaged into StemFlex supplemented with 2 μM thiazovivin and drug, with prexasertib (Chk1i) or adavosertib (Wee1i) added at 62 nM. At day 6, cells were replated and fresh drug was added to ensure removal of dead cells. At day 8, the fraction of sgRNA+ (BFP+) surviving cells were analyzed by flow cytometry.

For Fig. 4I, human iPSC line H21792A and chimpanzee iPSC C40280L were co-cultured with a 50/50 initial seeding density. After one normal passage, cells were dissociated with Accutase and passaged in StemFlex supplemented with 2 μM thiazovivin and drug, with prexasertib or adavosertib added at 125 nM. 24hr after drug treatment, cells were replated and fresh drug was added to ensure removal of dead cells. 48 hours post drug treatment, the ratio of human cells (GFP+) chimpanzee cells (mCherry+) was analyzed by flow cytometry.

#### FUCCI reporter live imaging

An updated version of the FUCCI cell cycle reporter was cloned into a lentiviral expression vector with a universal chromatin opening element (UCOE), an EF-1α promoter driving Cdt1 (1-100) C-terminal fused to mCherry, and a woodchuck hepatitis virus post-transcriptional regulatory element (WPRE)^77^. A four amino acid SGGS linker was used between mCherry and the Cdt1 fragment. This construct constitutes half of the FUCCI cell cycle reporter and has been reported to fluoresce red during G1 phase but is otherwise degraded by both SCF^Skp2^ and CUL4^Ddb1^ E3 ubiquitin ligases.

Human (21792A) and chimpanzee (3624K) cells were infected with the Cdt1 reporter. At day 5 post-infection, polyclonal populations of cells expressing the Cdt1 reporter were sorted based on mCherry expression on a Sony MA900. Reporter cells were then cross validated with the commercial CLICK-iT EdU cell cycle assay, with mCherry positive cells nearly all classified as G1 phase by the EdU assay (88%). Similarly, mCherry negative cells were nearly fully depleted from G1 phase (3%).

Next, Cdt1 reporter cells were infected with either an sgRNA targeting *CDK2* or a non-targeting sgRNA control, with 25-40% of cells expression sgRNAs based on GFP expression. At day 4 post-infection, cells were seeded onto an Ibidi 4-well μ-Slide, with one well for each condition. On day 5 post-infection, cells were live imaged on a Nikon Ti automated inverted microscope with incubation enclosure for a period of 24.83 hours at an interval of 10 minutes across 38 stage positions.

#### Neural progenitor cell differentiation

Human (28126B) and chimpanzee (40280L) PSCs were differentiated into neural progenitor cells (NPCs) as described in ^129^ with the following modifications. Differentiation media was made without ventral and caudalization patterning factors (sonic hedgehog agonist and GSK3i CHIR99021). PSCs were maintained on Matrigel (Corning, cat. 354230) prior to day 0 plating onto Lam-111 coated plates. Cells were seeded at 20,000 cells/cm^2^, as measured by Chemometec Nucleocounter NC-202, roughly twice the published density to ensure robust survival. NPCs were evaluated for purity at days 7-11 of differentiation with antibody staining against NPC markers Pax6 and Nestin. Cells were dissociated with Accutase, pelleted and washed, then fixed and permeabilized with the BD Cytofix/Cytoperm kit (ThermoFisher, cat. BDB554714). 100 μl cells were stained with 2 μl human anti-Pax6-APC (Miltenyi, cat. 130-123-328) + 5 μl mouse anti-Nestin-PE (Biolegend, cat. 656805) and evaluated by flow cytometry. In addition, NPCs were plated on μ-Slide 4 Well chambers (Ibidi cat. 80426), stained with antibodies against Pax6 and Nestin, and visualized by fluorescence microscopy on a RPI spinning disk confocal microscope.

#### Cerebral organoid differentiation

Human (H1) and Chimpanzee (8861G) cells were reverse transduced with lentiviral constructs containing sgRNAs targeting *CDK2*, *CDK4*, *CCNE1*, *RBL1*, *ATP6AP1*, *ASPM*, or a non-targeting sgRNA. An additional pair of human (23555A) and chimpanzee (40280L) organoids were transduced with lentiviral constructs containing sgRNAs targeting cell cycle factors *CDK2*, *CDK4*, *CCNE1*, *RBL1*, or a non-targeting sgRNA. 48 hours post-infection, cells expressing sgRNA were selected for via addition of 1.5 μg/ml puromycin. Cells were then recovered in normal growth media from 4 days post-infection to 6 days post-infection. At 6 days post-infection, each sgRNA condition was single cell dissociated with Accutase, counted on a Chemometec Nucleocounter NC-202, and seeded into an ultra low-attachment V-bottom 96 well plate (Prime Surface cat. MS-9096VZ). Four to twelve wells were seeded per condition with 10,000 cells seeded in 100 μl of Sasai Media #1.

Organoid formation was performed according to the protocol established by the Sasai lab^131^ with the following modifications. On days 0-6, wnt inhibitor Wnt-C59 was added to promote patterning to telencephalon. 200 nM LDN-193189 was added to promote differentiation from days 0 through 18.

#### Cerebral organoid EdU staining

Human (H1, 20961B, 23555A) and Chimpanzee (8861G and 40280L) cells were reverse transduced with lentiviral constructs containing sgRNAs targeting *CDK2* or a non-targeting sgRNA. 48 hours post-infection, cells expressing sgRNA were selected for via addition of 1.5 μg/ml puromycin. Only one day of puromycin selection was performed to maintain a sub-population (20-30%) of non-sgRNA expressing cells to enable co-culture analysis. Cells were then recovered in normal growth media from 3 days post-infection to 6 days post-infection. At 6 days post-infection, each sgRNA condition was dissociated to single cells with Accutase, counted on a Chemometec Nucleocounter NC-202, and seeded into an ultra low-attachment V-bottom 96 well plate (Prime Surface cat. MS-9096VZ). Twelve wells were seeded per replicate in each condition with 10,000 cells seeded in 100 μl of Sasai Media #1.

Organoids were collected on day 9 for cell cycle measurements to examine whether perturbations continue to influence cell cycle progression during NPC patterning and expansion. This time point was also chosen because several lines (20961B, 23555A, 8861G, and 40280L) exhibited significant silencing of the EF-1α promoter at day 18, which drives GFP expression within the sgRNA cassette. Some silencing was also apparent at day 9, with a bias for non-silenced cells to be actively proliferating, resulting in an apparent reduction of cells in G1 phase for the non-targeting sgRNA. Organoids were incubated in 10 μM EdU with a fresh media change and cells were incubated for roughly 1 hour, after which organoids were dissociated with a pre-warmed solution of Papain (Worthington Biochemical Corporation) mixed with DNase I according to manufacturer’s instructions. For single-cell dissociation, organoid samples were cut into small pieces, and incubated with Papain for 30 minutes. Cells were then pelleted and then directly fixed in 4% paraformaldehyde for 15 minutes at room temperature, protected from light. Cell cycle phase measurements were then performed as described above with the Click-iT Plus EdU Alexa Fluor 647 Flow Cytometry Assay Kit (ThermoFisher, cat. C10635).

#### Orangutan CRISPRi growth comparison

CRISPRi machinery was engineered into orangutan PSCs^107^ at the AAVS1 locus via the three plasmid lipofection method described above (see Construction of CRISPRi cell lines). To account for mutations in the orangutan genome, the sgRNA for the Cas9 nuclease component was modified to perfectly match the orangutan AAVS1 locus. However, flanking regions were not modified. sgRNAs targeting *CDK2*, *CDK4*, *CCNE1*, *ATP6AP1*, *KAT6A*, and *UFL1* were transduced into human (28126B), chimpanzee (40280L), and orangutan PSCs via lentivirus at MOI ~1. Cells were transduced in triplicate in 24-well plates and passaged every 2 days in StemFlex supplemented with 2 μM thiazovivin. At each passage, a portion of cells were quantified by flow cytometry on a BD LSRFortessa. The fraction of sgRNA+ expressing cells was determined based on the fraction of BFP+ cells. Measurements were collected until day 10 for *CDK2*, *CDK4*, *CCNE1*, and *ATP6AP1*. Measurements were collected until day 14 for *KAT6A* and *UFL1*. We chose to carry out 4 additional days of growth for *KAT6A* and *UFL1* because the growth differences between species were smaller for these genes compared to *CDK2*, *CDK4*, and *ATP6AP1*, and the additional depletion of sgRNA-expressing cells that occurred between days 10 to 14 enabled finer resolution of species differences. In parallel, cells were transduced with sgRNAs targeting *CDK2*, *CDK4*, *CCNE1*, *ATP6AP1*, *KAT6A*, and *UFL1* and expanded into a 6-well plate. 48h post-infection, cells were expanded in 2 μg/ml puromycin and allowed to recover for 48 hours. At day 5 post-infection, sgRNA+ cells were sorted based on BFP+ expression using a Sony MA900. RNA was extracted, cDNA was reverse transcribed, and qRT-PCR was used to quantify the degree of sgRNA-mediated depletion in biological triplicate (as described above in Quantitative RT-PCR).

### QUANTIFICATION AND STATISTICAL ANALYSIS

#### Data analysis for pooled genome-wide CRISPRi screens

Sequencing data were aligned to hCRISPRi-v2 and quantified using the ScreenProcessing pipeline (https://github.com/mhorlbeck/ScreenProcessing)^44^. sgRNA counts were then processed using MAGeCK^47^ test (--norm-method control --remove-zero control --gene-test-fdr-threshold 0.10 --remove-zero-threshold 50 --gene-lfc-method alphamean) and separately using a custom analysis method inspired by MAGeCK. Briefly, sgRNA counts were normalized by the median ratio method. Mean-variance modeling was performed with non-targeting sgRNAs as the control group, and sample mean and variance values were used to parameterize a negative binomial distribution. *P*-values were then calculated for each sgRNA based on the tail probability of the negative binomial and *P*-value cut-offs were chosen such that 95% of non-targeting sgRNAs were not significant.

For each gene, sgRNAs were filtered by two criteria: 1) perfect alignment to both the human (hg38) and chimpanzee (panTro6) reference genomes, as determined by FlashFry^58^, and 2) significance according to the negative binomial distribution. Only sgRNAs passing both filters were retained for analysis, resulting in a variable number of sgRNAs per gene (0-5 sgRNAs). The remaining sgRNA counts were converted to log_2_ fold-change and averaged to produce a gene score. Significance testing for gene scores was performed by bootstrapping non-targeting sgRNAs, with groups of 1-5 random non-targeting sgRNAs assigned to each control gene to select candidates from the initial genome-wide screens.

Essential genes for each screen (Fig. 1B) were determined by the degree of depletion among sgRNAs targeting the gene. To facilitate equal comparison among screens, the top 3000 most depleted genes in each screen were defined as essential (with mean sgRNA depletion greater than 4-fold for all such genes). In addition, genes with mean sgRNA depletion less than 2-fold were defined as non-essential. Each intersection set was then constructed based on two inclusion criteria: genes were required to be essential for each member of the set and non-essential for all non-members of the set.

For comparison of technical replicates (Fig. 1B), identification of shared essential genes (Fig. 1B), and assessment of screen performance using DepMap Public 21Q4 gene sets (Fig. 1C), all hCRISPRi-v2 sgRNAs, including those with mismatched targets in the panTro6 reference genome, were included for analysis. For identification of candidate genes with species-specific effects on proliferation (Fig. 1D), only sgRNAs with perfect-match targets in the panTro6 reference genome (77.4%, *n* = 79417/102640) and transcriptional start sites targeted by at least three sgRNAs after excluding mismatched sgRNAs (86.7%, *n* = 17804/20528), were retained for analysis.

For screen analysis using MAGeCK, genes with false discovery rates (FDRs) less than 10% for both individuals within a species and FDRs greater than 25% in both individuals from the opposite species were considered as candidates for validation screening (*n* = 418 genes).

#### CEV-v1 library selection criteria

Candidate genes for the CEV-v1 library were chosen based on the union of the bootstrapping method, MAGeCK analysis, and additional genes selected based on notable evolutionary history. The two methods were largely complementary. In total, we classified 339 candidate species-specific genetic dependencies with MAGeCK and 796 candidates with the more permissive bootstrapping method (57 candidate genes with “notable evolutionary history” were later added). By constructing the CEV-v1 validation library based on the union of these gene sets, we aimed to compile a permissive list of candidates to account for potential false negatives from the initial screens.

963 candidate species-specific essential or proliferation suppressor genes were selected for inclusion in the Comparative Essential Validation (CEV-v1) sgRNA library according to a series of criteria (Figure S3D).

First, genes were selected based on a combination of the magnitude of sgRNA enrichment or depletion and significance score from the custom bootstrapping analysis. We defined human_avg as the mean of the gene averaged log_2_FC for the two human PSCs and chimp_avg as the mean of the two chimpanzee PSCs.

Human essential set 1 was defined as: chimp_avg > −1 & chimp_avg < 1 & human_avg < −2 & human_avg - chimp_avg < −2 & bootstrap FDR < 0.05 in both of the human lines.

Human enriched set 1 was defined as: chimp_avg > −1 & chimp_avg < 1 & human_avg > 1 & human_avg - chimp_avg > 0.2 & bootstrap FDR < 0.05 in both of the human lines.

Chimpanzee essential set 1 was defined as: chimp_avg < −2 & human_avg < 1 & human_avg > −1 & human_avg - chimp_avg > 2 & bootstrap FDR < 0.05 in both of the chimpanzee lines.

Chimpanzee enriched set 1 was defined as: chimp_avg > 2 & human_avg < 1 & human_avg > −1 & human_avg - chimp_avg < −2 & bootstrap FDR < 0.05 in both of the chimpanzee lines.

Chimpanzee essential human enriched was defined as: chimp_avg < −0.8 & human_avg > 0.4 & human_avg - chimp_avg > 0.2 & bootstrap FDR < 0.05 in all four PSC lines.

Human essential chimp enriched was defined as: chimp_avg > 0.8 & human_avg < −0.8 & human_avg - chimp_avg < −0.2 & bootstrap FDR < 0.05 in all four PSC lines.

Both essential human more essential was defined as: chimp_avg < −1 & human_avg < −2.5 & human_avg/chimp_avg > 2 & bootstrap FDR < 0.05 in all four PSC lines.

Both essential chimp more essential was defined as: chimp_avg < −3 & human_avg < −1 & human_avg/chimp_avg < 0.3 & bootstrap FDR < 0.05 in all four PSC lines.

We added an additional permissive set of human and chimpanzee essential and enriched genes based not on the average magnitude of enrichment, which can be skewed by single sgRNAs with strong effects, but on the directional significance of the five sgRNAs. For these additional genes, we calculated a direction_score for the human and chimpanzee arms, defined as the number of significantly depleted sgRNAs targeting a gene minus the number of significantly enriched sgRNAs. Human_direction_score was defined as the sum of WTC11_direction_score + H1_ direction_score. Chimpanzee_direction_score was defined as the sum of C3649_direction_score + Pt5C_ direction_score. The additional gene sets were selected based on the following criteria:

Human essential set 2 was defined as: human_direction_score > 4 & WTC11_direction_score >= 2 & H1_direction_score >=2 & human_direction_score - chimp_direction_score >= 4.

Human enriched set 2 was defined as: human_direction_score < −4 & WTC11_direction_score <= −1 & H1_direction_score <=−2 & human_direction_score - chimp_direction_score <= −3 & log2(abs(human_direction_score/chimp_direction_score)) >= 1.

Chimp essential set 2 was defined as: human_direction_score > 4 & C3649_direction_score >= −1 & Pt5C_direction_score >=−2 & chimp_direction_score - human_direction_score >= 4 & log2(abs(human_direction_score/chimp_direction_score)) >= 1.

Chimp enriched set 2 was defined as: chimp_direction_score < −4 & C3649_direction_score <=− 2 & Pt5C_direction_score <=−2 & chimp_direction_score - human_direction_score <= −4 & log2(abs(human_direction_score/chimp_direction_score)) >= 1.

For MAGeCK, genes with false discovery rates (FDRs) less than 10% for both individuals within a species and FDRs greater than 25% in both individuals from the opposite species were considered as candidates for validation screening (*n* = 418 genes). Candidate genes nominated by MAGeCK were intersected with the gene sets listed above.

Lastly, a total of 57 candidate genes in the CEV-v1 validation library were selected based on notable evolutionary history. Of these, 42/57 covered previously annotated human-specific gene duplication events^56,143^ that were targetable by unique sgRNAs. These gene duplications included genes such as ARHGAP11A/B and NOTCH2/2NL, which have been implicated in the expansion of the human cortex^9-12^. The remaining 15/57 genes were approaching significance and were selected based on gene function, such as AGO1/2 and DROSHA, which we hypothesized to be involved in repression of transposable retroelements expressed in pluripotent stem cells.

#### Data analysis for CEV-v1 validation screens

Sequencing data were aligned to CEV-v1 and quantified using the ScreenProcessing pipeline and MAGeCK. A matrix containing sgRNA counts from all CEV-v1 screens (excluding C3649 and Pt5-C due to non-responsiveness to *MDM2*/*TP53* perturbations) was assembled and used as input for differential analysis by DESeq2. Briefly, each sample was annotated by species, individual, and timepoint and a design matrix was created to model the species-specific effect of time (t_0_ vs. t_final_) while controlling for individual effects (modeled as fixed effects) within each species. The human and chimpanzee species terms were then contrasted to extract a Benjamini–Hochberg-adjusted *P-*value and log_2_ fold-change for each sgRNA. sgRNA adjusted *P-*values were combined into gene FDRs using alpha-robust rank aggregation (α-RRA) from MAGeCK and the α threshold (to remove the effect of insignificant sgRNAs from the assessment of gene significance) was set according to the fraction of sgRNAs with an adjusted *P-*value < 0.01. For each gene, log_2_ fold-change was computed as the mean of the four sgRNAs with the largest absolute fold-change. To exclude genes with shared effects from being erroneously called as species-specific, any gene with an FDR in both the human and chimpanzee species terms less than the highest FDR for any gene with at least one sgRNA passing the α threshold in α-RRA was discarded. For each gene in the set of 75 genes with species-specific effects reported in Fig. 2, we required that three conditions be met in the chimpanzee–human contrast term: (1) gene FDR < 0.01, (2) at least three sgRNAs targeting the gene pass the α threshold in α-RRA, and (3) gene log_2_ fold-change difference ≥ 0.75 between species. We used the STRING database v11.5^71^ to identify known and predicted protein–protein interactions among this set of 75 genes.

To quantify sources of variation in CEV-v1 screens, a matrix of sgRNA counts was assembled as described above and normalized using edgeR^144^ calcNormFactors. Normalized sgRNA counts were then prepared for linear modeling using variancePartition voomWithDreamWeights and a linear mixed model was fit using variancePartition fitExtractVarPartModel. The categorical variables species, individual, and timepoint were modeled as random effects. Because most individuals were only screened once, the individual term describes variation attributable to differences between independent screens as well as differences between individuals. For each gene (Fig. S2B), gene-level estimates of variance were determined by computing the mean variance attributable to each variable for all sgRNAs targeting that gene.

Potential p53-dependent candidate genes were flagged according to two methods. First, each gene was cross-referenced to previous growth screens performed in eight p53 wild-type and six p53 mutant AML cell lines^145^. Second, the two p53-unresponsive chimpanzee cell lines were compared to the four p53-responsive chimpanzee cells with a two-tailed t-test. Results from both lines of analysis are presented in Table S4C. Gene ontology enrichments of p53-dependent candidate gene set is presented in Table S5.

#### Analysis of protein-coding and gene expression changes

To obtain coding sequences for homologous transcripts from human and chimpanzee reference genomes, we downloaded human protein and transcript sequences from Gencode release 36^146^ and chimpanzee protein and transcript sequences from the Comparative Annotation Toolkit^147^ annotation on reference version panTro6 produced as part of a recent study^141^. For each human transcript of each protein coding gene, we obtained the transcript sequence and its canonical translation, and we then extracted the corresponding chimpanzee transcript and canonical translation by matching the Ensembl transcript ID to its chimpanzee counterpart (“source transcript” field in the chimpanzee gene annotation). For both the human and chimpanzee sequence of each transcript, we then compared the translated sequence at every possible start codon and frame to the canonical amino acid sequence, determining the start codon and frame that produced the canonical amino acid sequence to be “correct” and removing bases thus found to belong to the 5’ or 3’ UTR (upstream of the correct start codon or downstream of the correct stop codon).

With coding sequences for homologous transcripts, we then aligned the human and chimpanzee protein sequences using the pairwise2 module from Biopython^148^ with a BLOSUM62 substitution matrix. We then deleted codons in transcripts corresponding to amino acids that aligned to a gap in the other amino acid sequence. Finally, we deleted stop codons from the ends of sequences. We then wrote out each pairwise alignment to a control file for PAML^149^ and calculated relevant statistics, including dN, dS, N, and S, using PAML’s implementation of the Yang and Nielson 2000 (yn00) algorithm^150^. Finally, to avoid undefined values, we set dS to 1/S where dS was zero and selected the median dN value and median dN/dS value per gene for analysis.

Distributions of dN and dN/dS were compared for the full set of genes, DepMap common essential genes, and validation screen hits by two-sided Kolmogorov-Smirnov test (ks.test in R).

#### RNA-seq analysis

Raw bulk RNA-seq reads from knockdown experiments and wild-type chimpanzee and human iPSCs were adapter-trimmed using cutadapt^125^ (with option -b AGATCGGAAGAGCACACGTCTGAACTCCAGTCA) and then pseudo-aligned to species-specific transcriptomes using kallisto^126^ with options --single -l 200 -s 20. Transcriptomes were extracted from species-specific gtf annotations using the gffread utility^127^ using the -w output option. Human transcripts were obtained from the Gencode^146^ comprehensive gene annotation v36 (GTF), using genome assembly hg38^151^, and the chimpanzee annotation was obtained from a recent study that produced a hierarchical alignment of primate genome assemblies^141^ and annotated the assemblies using the Comparative Annotation Toolkit^147^.

To ensure consistency of gene names across the annotations, we downloaded the set of gene aliases from the HUGO Gene Nomenclature Committee website (www.genenames.org; ^140^ and searched for gene names present in the chimpanzee but missing in the human annotation, mapped to aliases present in the human annotation. This led us to rename five genes in the non-human primate annotations (DEC1 to *BHLHE40*, *DUSP27* to *DUSP29*, *AC073585.2* to *FAM24B*, *LOR* to *LOXL2*, and *TNRC6C-AS1* to *TMC6*); we also renamed *CCNP* in the human annotation to *CNTD2*.

After counting transcript abundances using kallisto, we converted them to gene counts using the tximport command in the tximport R package^128^ with the options type=‘kallisto’ and countsFromAbundance=‘no’. We then created one human and one chimpanzee data set in DESeq2^59^ contrasting gene knockdowns with wild-type gene expression in two replicates of the same cell line in each. We extracted VST-transformed counts for plotting using the function vst with option blind=TRUE and ran the DESeq linear model fitting using the function DESeq with betaPrior=TRUE.

#### FUCCI reporter live analysis

Images were pre-processed in MATLAB to rescale intensity to fit the full dynamic range of the image. Due to variable illumination at different stage positions, manual adjustments to the intensity range were made to minimize background signal and to enable accurate segmentation of mCherry-Cdt1+ nuclei.

Image segmentation was performed using a CellProfiler^152^ pipeline with the following steps: 1) IdentifyPrimaryObjects, 2) MeasureObjectSizeShape, 3) MeasureObjectIntensity, 4) ConvertObjectsToImage, 5) TrackObjects, 6) SaveImages, 7) ExportToSpreadsheet. All image segmentation was performed in the mCherry channel, though GFP fluorescence was measured for each segmented object to determine sgRNA status. Primary cell segmentation was performed with a typical diameter range of 8 to 60 pixels and a threshold correction factor of 3. Thresholding was performed with the global minimum cross-entropy method. Nuclei were then tracked from one time point to the next with the overlap method, with a maximum distance to consider matches of 40 pixels.

Further post-processing refinement of the nuclei tracking step was performed to match nuclei that had moved enough during the 10 minute imaging interval to have zero overlap with the previous timepoint, but likely represented the same cell based on spatial proximity. For nuclei that were unassigned based on the CellProfiler TrackObjects module, we calculated the Euclidean distance to all nuclei in the previous frame based on the centroids of each object. If the unassigned nucleus was within 25 pixels of a nucleus from the previous that did not already have a matched nucleus in the current frame, then we reassigned both nuclei to the same label. We did not consider the possibility of nuclei splitting into two daughter cells, as the mCherry-Cdt1 reporter fluorescence is restricted to G1 phase, and thus nuclei were tracked to a single object in each frame.

We measured the lifetime of each nucleus based on the number of frames in which the nucleus could be segmented and tracked within our time series. Nuclei present at the initial time point or the final time point were filtered, as its lifetime could extend for an unknown period beyond the start or end of the experiment. In addition, nuclei with lifetime shorter than 30 minutes or 6 hours were filtered out, as these nuclei likely represent extremes in overall reporter expression that may confound accurate measurement of G1-phase length. Lastly, nuclei were filtered based on area, with a minimum area of 60 square pixels and a maximum area of 550 square pixels. GFP was quantified as the average GFP intensity within the segmented nucleus, averaged across the entire time course of the object’s lifetime. sgRNA expressing cells were classified as cells with mean GFP intensity greater than 0.15 (on a 0 to 1 scale) and sgRNA non-expressing cells within the same well were classified as cells with intensity less than 0.02. The distributions of nuclei lifetimes were then visualized on violin plots for each cell line and sgRNA perturbation.

#### Cerebral organoid brightfield image analysis

Brightfield images were captured on a EVOS M5000 microscope with a 4x objective. Day 18 was selected as a measurement time point because after 18, organoids are transferred from individual wells of a 96 well plate to 6-well petri dishes, allowing for standardized imaging. For a small number of conditions (40280L sgCDK2 and sgCCNE1), a fraction of organoids disaggregated due to cell death before day 18 and size measurements were not collected.

Images were first manually pre-processed in ImageJ to maximize contrast between organoid edges and background debris. Contrast adjusted images were then quantified with a CellProfiler^152^ pipeline with the following steps: 1) IdentifyPrimaryObjects, 2) MeasureObjectSizeShape, 3) ConvertObjectsToImage, 4) SaveImages, 5) ExportToSpreadsheet. Organoid segmentation was performed with a typical diameter range of 150 to 600 pixels and a threshold correction factor of 1. Thresholding was performed with the global minimum cross-entropy method. Organoid segmentation was manually checked for accuracy and absence of multiple objects.

## Supplementary Material

Figure S2 Figure S2. CEV-v1 validation screens in human and chimpanzee PSCs, Related to Figure 2.
(A) Bulk RNA-seq VST-transformed counts for key pluripotency markers Oct4, Sox2, and Nanog across CRISPRi engineered PSCs from this study and original source lines. (B) Principal component analysis of bulk RNA-seq transcriptomes, with PC1 capturing batch effects between two separate sequencing experiments and PC2 capturing human and chimpanzee species differences. (C) CaSpER analysis of chromosomal copy number variations from bulk RNA-seq data across all newly engineered CRISPRi PSC lines as aligned to the human hg38 reference genome. For the chimpanzee genome, chromosome 2 refers to 2a and 2b. (D) Distribution of positions relative to the FANTOM-annotated TSS for sgRNAs in CEV-v1. Vertical colored lines indicate the selection round in which sgRNAs were chosen. (E) Distribution of DeepHF on-target predictions for sgRNAs in CEV-v1. (F) Selection criteria for CEV-v1 sgRNA library. (G) Inclusion criteria for 963 genes selected as candidate species-specific genetic dependencies.

Figure S1 Figure S1. Genome-wide CRISPRi screens in human and chimpanzee PSCs, Related to Figure 1
(A) Log_2_ fold-change of sgRNA counts from genome-wide CRISPRi screens using the hCRISPRi-2 sgRNA library, averaged across two human and two chimpanzee cell lines. sgRNAs containing mismatches to the chimpanzee genome are colored in red and non-targeting sgRNAs are colored in yellow. Thus, a substantial number of mismatched sgRNAs targeting essential genes are depleted in human PSCs but not in chimpanzee PSCs. (B) Depletion or enrichment of sgRNA counts at growth day 10 compared to growth day 0. Non-targeting sgRNAs are colored in yellow, and sgRNAs characterized as non-significant by mean-variance modeling of a negative binomial than 2-fold and FDR < 0.05 for each individual screen. Average log_2_ fold-change for BAK1 sgRNAs for each individual screen. (D) Averaged species-level gene log_2_ fold-change for apoptosis related genes and p53 related genes. (E) Heatmap displaying log_2_ fold-change of sgRNA counts across five human and six chimpanzee PSCs, with columns 1, 6, and 7 showing primary genome-wide screening data and remaining columns showing data from secondary validation screening. Columns 6 and 7 (Pt5-C and C3649) represent the two chimpanzee PSCs that exhibit TP53 mutant phenotypes. (F) UpSet plot showing the intersection of candidate p53-dependent essential genes across all four screens. Gene ontology (GO) enrichment terms for the set of 127 candidate p53-dependent genes.

Figure S7 Figure S7. Tri-species comparison of human, chimpanzee, and orangutan PSCs expressing sgRNAs targeting *CCNE1* and *DDRGK1*, Related to Figure 7.
(A) qRT-PCR measurements of sgRNA knockdown efficiency for sgCDK2, measured with an alternative primer set. (B-C) Change in the relative fraction of CCNE1 (B) and DDRGK1 (C) sgRNA containing cells over time in human (28126B), chimpanzee (40280L), and orangutan PSCs. qRT-PCR measurements of sgRNA knockdown efficiency for each sgRNA.

Figure S6 Figure S6. Derivation of human and chimpanzee NPCs, Related to Figure 5.
(A) Chimpanzee neural progenitor cells (40280L) stained for Pax6 and Nestin, visualized by confocal microscopy. (B) Chimpanzee neural progenitor cells (40280L) stained for Pax6 and Nestin, quantified by flow cytometry. (C) Negative control PSCs stained for Pax6 and Nestin, quantified by flow cytometry. (D) Organoid size measurements for human (23555A) and chimpanzee (40280L) cerebral organoids, measured on day 18 by brightfield microscopy (N=3-12). Bar charts plotted as mean ± s.e.m, with each individual data point representing an independent organoid.

Figure S5 Figure S5. Measurements of species differences in G1 phase length, Related to Figure 4.
(A) Absolute fraction of human (21792A and 28126B, blue), chimpanzee (3624K, 40280L, and 8861G, red) PSCs in G1 phase, as measured by EdU incorporation and DNA content. Histogram of G1 fraction for both human and chimpanzee cells (black). (B) Relative fraction of cells in G1 phase for six pairs of human and chimpanzee PSCs co-cultured in the same well (N=4). (C) Cell viability measurements for chimpanzee PSCs (40280L) expressing sgRNAs targeting cell cycle regulators (N=3, s.e.m.). (D) FUCCI reporter cell line cross validation with cell cycle proportion measurements via EdU incorporation and DNA content. (E) Quantification of G1 phase length by live imaging of human (21792A) and chimpanzee (3624K) PSCs infected with either an sgRNA targeting *CDK2* or a non-targeting sgRNA. (F) Whole-genome shotgun sequence coverage at all genes in the HUGO Gene Nomenclature Committee gene groups cyclins, cyclin dependent kinases, and class III Cys-based CDC25 phosphatases (https://www.genenames.org/). Each violin represents the coverage at each base across the entire body for each gene. The horizontal lines correspond to the 5th, 50th, and 95th percentiles of baseline coverage across the entire genome (“Genome” panel) or the X chromosome (“chrX” panel). Four genes in these sets located on chromosome X (*CDKL5*, *CDK16*, *CCNB3*, *CCNQ*) are shown separately to account for different baseline coverage; these gene names are outlined in a gray box in the legend. The top three rows correspond to chimpanzee PSCs from individuals used in the present study (C40280L, C8861G, C3649K), while the bottom three rows correspond to similarly reprogrammed chimpanzee individuals. (G) Strip plots of log_2_ fold-change for sgRNAs targeting *RB1* with data derived from only from primary genome-wide screen. Computationally predicted specificity score and off-target counts for each of the five sgRNAs targeting *RB1*^137^.

Figure S4 Figure S4. Species-specific genetic dependencies interact in biological processes and complexes, Related to Figure 3.
(A) dN, the rate of non-synonymous substitutions in a gene, and (B) dN/dS values, the ratio between the rates of non-synonymous and synonymous substitutions, for 75 validated differential-essentiality genes from this study compared to all genes or essential genes. (C) Comparative gene expression levels between human and chimpanzee PSCs for 75 validated differential-essentiality genes from this study vs. all genes expressed in PSCs. (D) sgRNA depletion or enrichment for all active sgRNAs targeting members of the UFMylation pathway, MOZ histone acetylation complex, *RBL1*, and the PAN2/3 complex. Each circle represents the sgRNA log_2_ fold-change for one sgRNA in one human (blue) or chimpanzee (red) individual. Each strip plot contains a variable number of columns, corresponding to the number of significant sgRNAs targeting each gene. Genes with only one significant sgRNA (*ING5* and *RBL1*) are scored as less significant compared to genes with multiple significant sgRNAs and require validation of on-target effects. (E) Co-culture of human PSCs (28126B) and chimpanzee PSCs (40280L) with wild-type cells (BFP−) and cells expressing sgATP6AP1 (BFP+) stained with LysoSensor Green and LysoTracker Red. Scale bar = 20 μm. (F) Western blot for phospho-S6 (pS6) expression and GAPDH loading control for one additional human (21792A) and one chimpanzee (8861G) cell line depleted for *ATP6AP1* with a non-targeting sgRNA control.

Figure S3 Figure S3. 75 genes with robust species-specific effects on cellular proliferation, Related to Figure 2.
(A) Strip plots of log_2_ fold-change in sgRNA enrichment or depletion for 75 genes with species-specific effects on cellular proliferation (FDR < 1%), calculated by α-RRA, colored by individual.

Table S4 **Table S4: Primary data for comparative essentiality validation screens, related to** Figure 2. (A) Log2FC for individual sgRNAs from CEV-v1 validation screens (B). CEV-v1 sgRNA library. (C) Gene averaged depletion scores for CEV-v1 validation screens, related to Figure 2.

Table S3 Table S3: Primers used in this study, related to Figure 1.

Table S1 Table S1: Cellular metadata for primary CRISPRi screen and CEV-v1 screens, related to Figure 1.

Table S5 Table S5. Gene Ontology (GO) biological process enrichments for candidate p53-dependent genes, related to Figure 2.

Table S2 **Table S2: Primary data for genome-wide screens, related to** Figure 1. (A) sgRNA counts from hCRISPRi-v2 genome-wide screens. (B) Custom bootstrap analysis for hCRISPRi-v2 genome-wide screens. (C) MAGeCK analysis results for hCRISPRi-v2 genome-wide screens. (D) hCRISPRi-v2 sgRNA library.

Video S1 Video S1. FUCCI reporter live imaging for 21792A sgNT, 21792A sgCDK2, 3624K sgNT, and 3624K sgCDK2, related to Figure 4.

## Figures and Tables

**Table:** KEY RESOURCES TABLE

REAGENT or RESOURCE	Source	Identifier
Antibodies		
anti-pS6, rabbit, 1:1,000	Cell Signaling	Cat# 2211S; RRID: AB_331679
anti-GAPDH, rabbit, 1:1,000	Proteintech	Cat# 10494-1-AP; RRID: AB_2263076
anti-PAX6, human, 1:50	Miltenyi	Cat# 130-123-328; RRID: AB_2819477
anti-B2M, mouse, 1:20	BioLegend	Cat# 316312; RRID: AB_10641281
anti-Nestin, mouse, 1:20	BioLegend	Cat# 656805; RRID: AB_2566381
Goat anti-Rabbit IGG secondary IRDye 680RD, 1:15,000	LI-COR	Cat# 926-68071; RRID: AB_10956166
Goat anti-Rabbit IGG secondary IRDye 800CW, 1:15,000	LI-COR	Cat# 926-32211; RRID: AB_621843
Bacterial and virus strains		
MegaX Competent Cells	ThermoFisher	Cat# C640003
Stellar Competent Cells	Takara	Cat# 636766
Chemicals, peptides, and recombinant proteins		
TransIT-LT1 Transfection Reagent	Mirus Bio	Cat# MIR2300
Lipofectamine Stem Transfection Reagent	ThermoFisher	Cat# STEM00008
SB431542	Miltenyi	Cat# 130-106-543
Noggin	Miltenyi	Cat# 130-103-456
ROCK inhibitor thiazovivin	Stem Cell Tech.	Cat# 72252
Puromycin	Goldbio	Cat# P-600-100
Chroman 1	Tocris	Cat# 7163
Emricasan	Selleck Chemicals	Cat# S7775
Polyamine Supplement	Sigma	Cat# P8483
Trans-ISRIB	Tocris	Cat# 5284
Nutlin-3a	Selleck Chemicals	Cat# S8059-5mg
Prexasertib	ThermoFisher	Cat# 501364492
Adavosertib	Selleck Chemicals	Cat# S1525-5mg
Knockout Serum Replacement	ThermoFisher	Cat# 10828028
G-MEM	ThermoFisher	Cat# 11710035
MEM NEAA	ThermoFisher	Cat# 11140050
Sodium Pyruvate	ThermoFisher	Cat# 11360070
β-mercaptoethanol	ThermoFisher	Cat# 31350010
N-2 supplement	ThermoFisher	Cat# A1370701
CD Lipid Concentrate	ThermoFisher	Cat# 11905031
Wnt-C59	ThermoFisher	Cat# 501493076
LDN-193189 Dihydrochloride	ThermoFisher	Cat# 605310
Amphotericin B	ThermoFisher	Cat# 15290018
Critical commercial assays		
Macherey-Nagel NucleoSpin Blood XL	ThermoFisher	Cat# NC1105387
Superscript IV VILO	ThermoFisher	Cat# 11756050
DyNAmo ColorFlash SYBR Green	ThermoFisher	Cat# F416L
Click-iT Plus EdU Alexa Fluor 647 Flow Cytometry Assay	ThermoFisher	Cat# C10635
Papain	Worthington	Cat# LK003150
BD Cytofix/Cytoperm	ThermoFisher	Cat# BDB554714
Experimental Models: Cell lines		
Chimpanzee iPSC 3624K dCas9-KRAB-mCherry	This paper	N/A
Chimpanzee iPSC 3624K dCas9-KRAB-BFP	This paper	N/A
Chimpanzee iPSC C3649 dCas9-KRAB-BFP	This paper	N/A
Chimpanzee iPSC 40290F dCas9-KRAB-mCherry	This paper	N/A
Chimpanzee iPSC 8861G dCas9-KRAB-mCherry	This paper	N/A
Chimpanzee iPSC 8861G dCas9-KRAB-BFP	This paper	N/A
Chimpanzee iPSC Pt-5c dCas9-KRAB-BFP	This paper	N/A
Human iPSC 20961B dCas9-KRAB-BFP	This paper	N/A
Human iPSC 20961B dCas9-KRAB-GFP	This paper	N/A
Human iPSC 21792A dCas9-KRAB-BFP	This paper	N/A
Human iPSC 23555A dCas9-KRAB-BFP	This paper	N/A
Human iPSC 23555A dCas9-KRAB-GFP	This paper	N/A
Human iPSC 28126B dCas9-KRAB-BFP	This paper	N/A
Human iPSC 28126B dCas9-KRAB-GFP	This paper	N/A
Human ESC H1 dCas9-KRAB-BFP	This paper	N/A
Human iPSC WTC11 dCas9-KRAB-BFP	Tian et al.^40^	N/A
Orangutan iPSC dCas9-KRAB-mCherry	This paper	N/A
Oligonucleotides		
See Table S3 for oligonucleotides used in this study	This paper	N/A
Recombinant DNA		
pX458	Ran et al.^121^	Addgene cat# 48138
Gen3-AAVS1 dCas9-XTEN-KRAB-P2A-BFP	Mandegar et al.^122^	Addgene cat# 73499
pC13N-dCas9-BFP-KRAB	Tian et al.^40^	Addgene cat# 127968
pEF1-BCL-XL	Li et al.^123^	
pCRISPRia-v2	Adamson et al.^118^	Addgene cat# 84832
pZT-C13-L1	Cerbini et al.^38^	Addgene cat# 62196
pZT-C13-R1	Cerbini et al.^38^	Addgene cat# 62197
pHR-UCOE-SFFV-dCas9-XTEN80-KRAB(Kox1)-P2A-EGFP	Replogle et al.^124^	Addgene cat# 188765
CDK1 cDNA clone 5873	Horizon Discovery	Cat# OHS1770-202320538
UCOE_EF1_mCherry hCDT1 1-100	This paper and Sakaue-Sawano et al.^77^	N/A
Deposited Data		
RNA-seq counts for human and chimpanzee wild-type iPSCs	Gallego Romero et al.^15^	https://elifesciences.org/articles/07103
RNA-seq counts for human and chimpanzee CRISPRi iPSCs	This paper	GSE212297
P53 wild-type and mutant AML growth screens	Wang et al.^145^	https://www.sciencedirect.com/science/article/pii/S0092867417300612
dN/dS scores for human protein coding genes	Dumas et al.^66^	https://genome.cshlp.org/content/31/3/484.long
Software and Algorithms		
CRISPRi screen processing for sgRNA counts	Horlbeck et al.^44^	https://github.com/mhorlbeck/ScreenProcessing
MaGeCK	Li et al.^47^	https://sourceforge.net/projects/mageck/
FlashFry	McKenna et al.^58^	https://github.com/mckennalab/FlashFry
Cutadapt	Martin, M.^125^	https://cutadapt.readthedocs.io/en/stable/
kallisto	Bray et al.^126^	https://pachterlab.github.io/kallisto/
gffread	Pertea et al.^127^	https://github.com/gpertea/gffread
tximport	Soneson et al.^128^	https://bioconductor.org/packages/release/bioc/html/tximport.html
DESeq2	Love et al.^59^	https://bioconductor.org/packages/release/bioc/html/DESeq2.html
