# Supplementary figures and images for: Comparative landscape of genetic dependencies in human and chimpanzee stem cells
Source: Cell. Author manuscript; Available in PMC 2024 Jul 6. (PMC10461406; doi:10.1016/j.cell.2023.05.043)

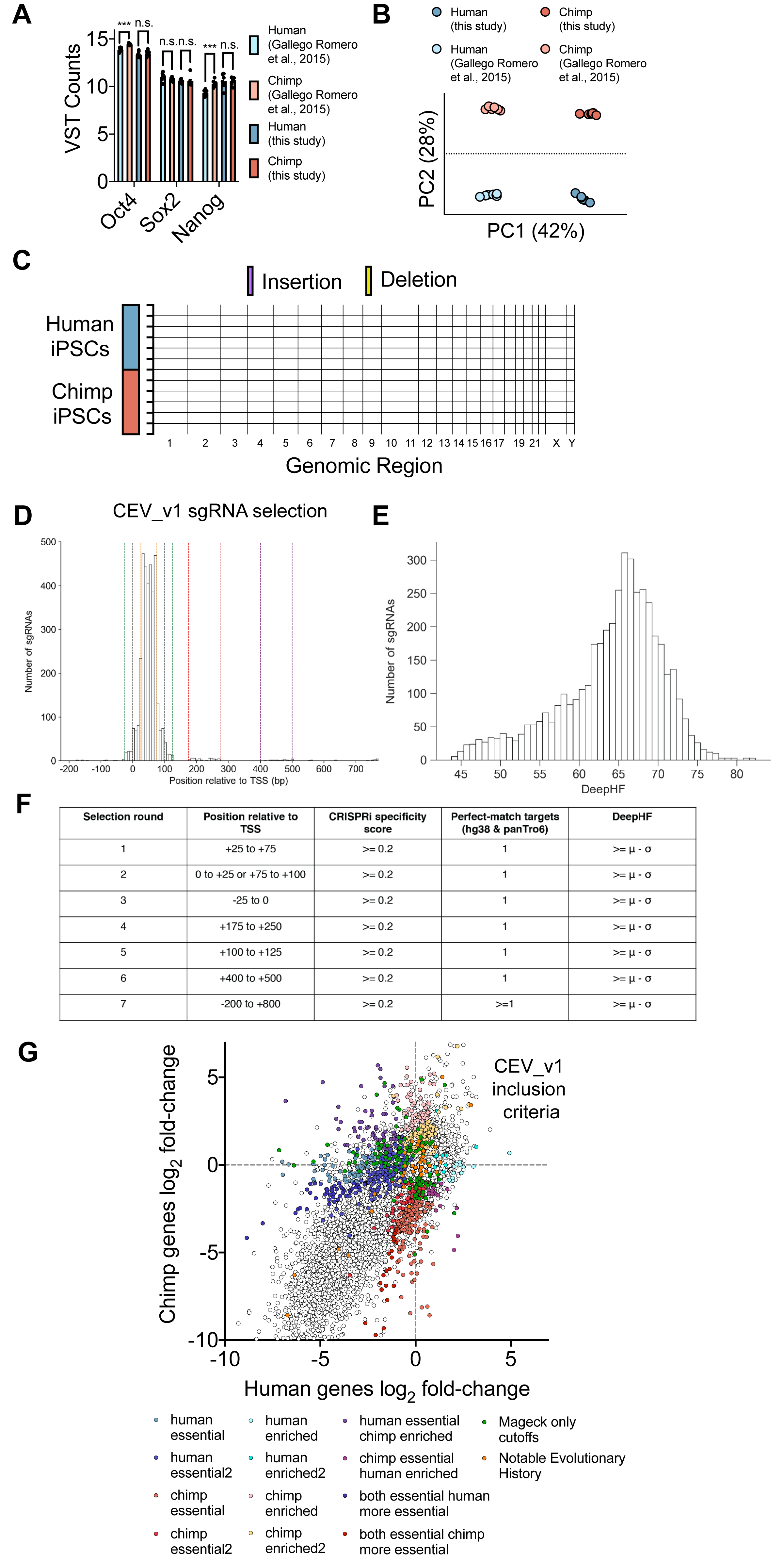

Supplement: Figure S2 — CEV-v1 validation screens in human and chimpanzee PSCs, Related to Figure 2. (A) Bulk RNA-seq VST-transformed counts for key pluripotency markers Oct4, Sox2, and Nanog across CRISPRi engineered PSCs from this study and original source lines. (B) Principal component analysis of bulk RNA-seq transcriptomes, with PC1 capturing batch effects between two separate sequencing experiments and PC2 capturing human and chimpanzee species differences. (C) CaSpER analysis of chromosomal copy number variations from bulk RNA-seq data across all newly engineered CRISPRi PSC lines as aligned to the human hg38 reference genome. For the chimpanzee genome, chromosome 2 refers to 2a and 2b. (D) Distribution of positions relative to the FANTOM-annotated TSS for sgRNAs in CEV-v1. Vertical colored lines indicate the selection round in which sgRNAs were chosen. (E) Distribution of DeepHF on-target predictions for sgRNAs in CEV-v1. (F) Selection criteria for CEV-v1 sgRNA library. (G) Inclusion criteria for 963 genes selected as candidate species-specific genetic dependencies. [file NIHMS1920094-supplement-Figure_S2.tif]

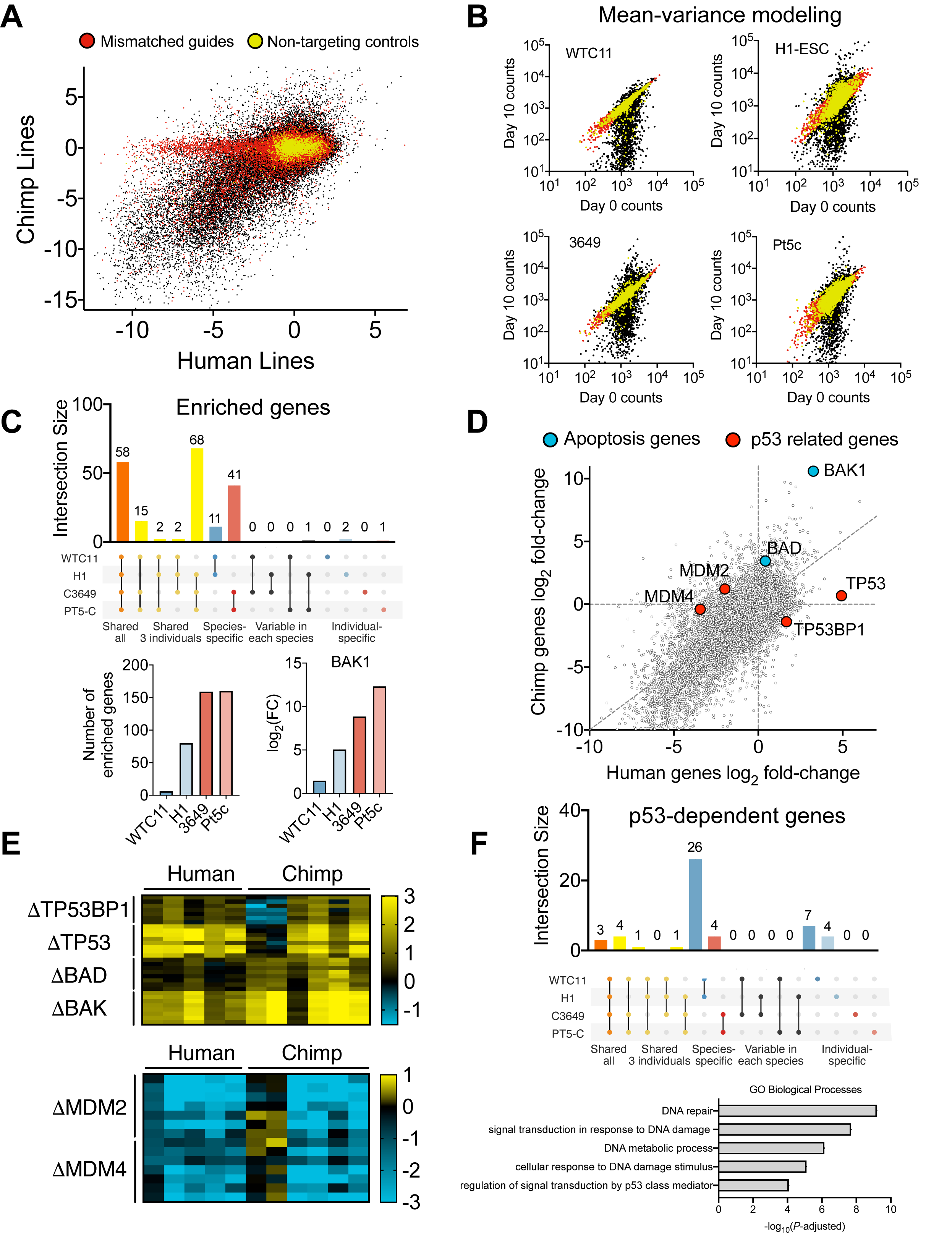

Supplement: Figure S1 — Genome-wide CRISPRi screens in human and chimpanzee PSCs, Related to Figure 1 (A) Log2 fold-change of sgRNA counts from genome-wide CRISPRi screens using the hCRISPRi-2 sgRNA library, averaged across two human and two chimpanzee cell lines. sgRNAs containing mismatches to the chimpanzee genome are colored in red and non-targeting sgRNAs are colored in yellow. Thus, a substantial number of mismatched sgRNAs targeting essential genes are depleted in human PSCs but not in chimpanzee PSCs. (B) Depletion or enrichment of sgRNA counts at growth day 10 compared to growth day 0. Non-targeting sgRNAs are colored in yellow, and sgRNAs characterized as non-significant by mean-variance modeling of a negative binomial than 2-fold and FDR < 0.05 for each individual screen. Average log2 fold-change for BAK1 sgRNAs for each individual screen. (D) Averaged species-level gene log2 fold-change for apoptosis related genes and p53 related genes. (E) Heatmap displaying log2 fold-change of sgRNA counts across five human and six chimpanzee PSCs, with columns 1, 6, and 7 showing primary genome-wide screening data and remaining columns showing data from secondary validation screening. Columns 6 and 7 (Pt5-C and C3649) represent the two chimpanzee PSCs that exhibit TP53 mutant phenotypes. (F) UpSet plot showing the intersection of candidate p53-dependent essential genes across all four screens. Gene ontology (GO) enrichment terms for the set of 127 candidate p53-dependent genes. [file NIHMS1920094-supplement-Figure_S1.tif]

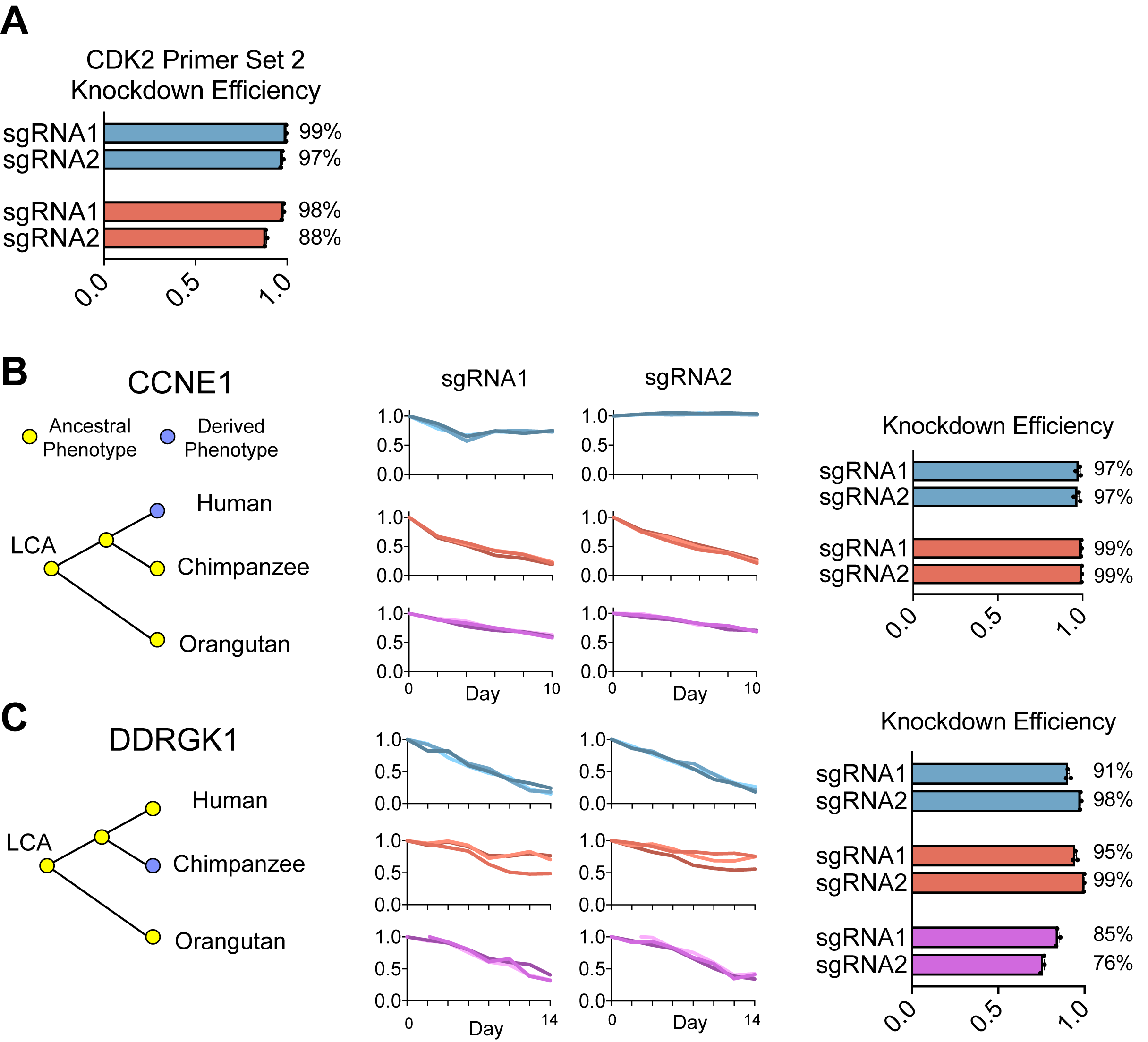

Supplement: Figure S7 — Tri-species comparison of human, chimpanzee, and orangutan PSCs expressing sgRNAs targeting CCNE1 and DDRGK1, Related to Figure 7. (A) qRT-PCR measurements of sgRNA knockdown efficiency for sgCDK2, measured with an alternative primer set. (B-C) Change in the relative fraction of CCNE1 (B) and DDRGK1 (C) sgRNA containing cells over time in human (28126B), chimpanzee (40280L), and orangutan PSCs. qRT-PCR measurements of sgRNA knockdown efficiency for each sgRNA. [file NIHMS1920094-supplement-Figure_S7.tif]

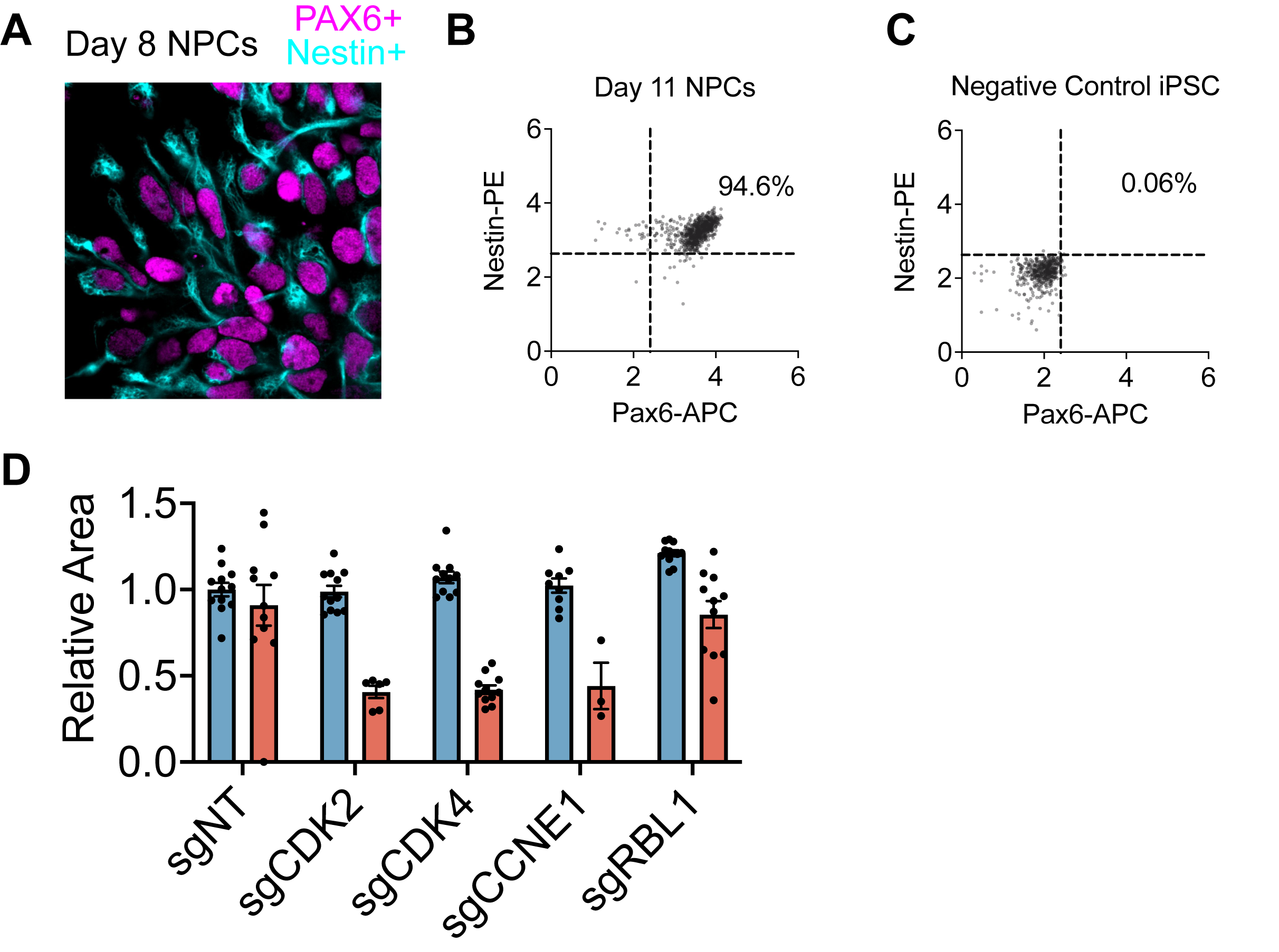

Supplement: Figure S6 — Derivation of human and chimpanzee NPCs, Related to Figure 5. (A) Chimpanzee neural progenitor cells (40280L) stained for Pax6 and Nestin, visualized by confocal microscopy. (B) Chimpanzee neural progenitor cells (40280L) stained for Pax6 and Nestin, quantified by flow cytometry. (C) Negative control PSCs stained for Pax6 and Nestin, quantified by flow cytometry. (D) Organoid size measurements for human (23555A) and chimpanzee (40280L) cerebral organoids, measured on day 18 by brightfield microscopy (N=3-12). Bar charts plotted as mean ± s.e.m, with each individual data point representing an independent organoid. [file NIHMS1920094-supplement-Figure_S6.tif]

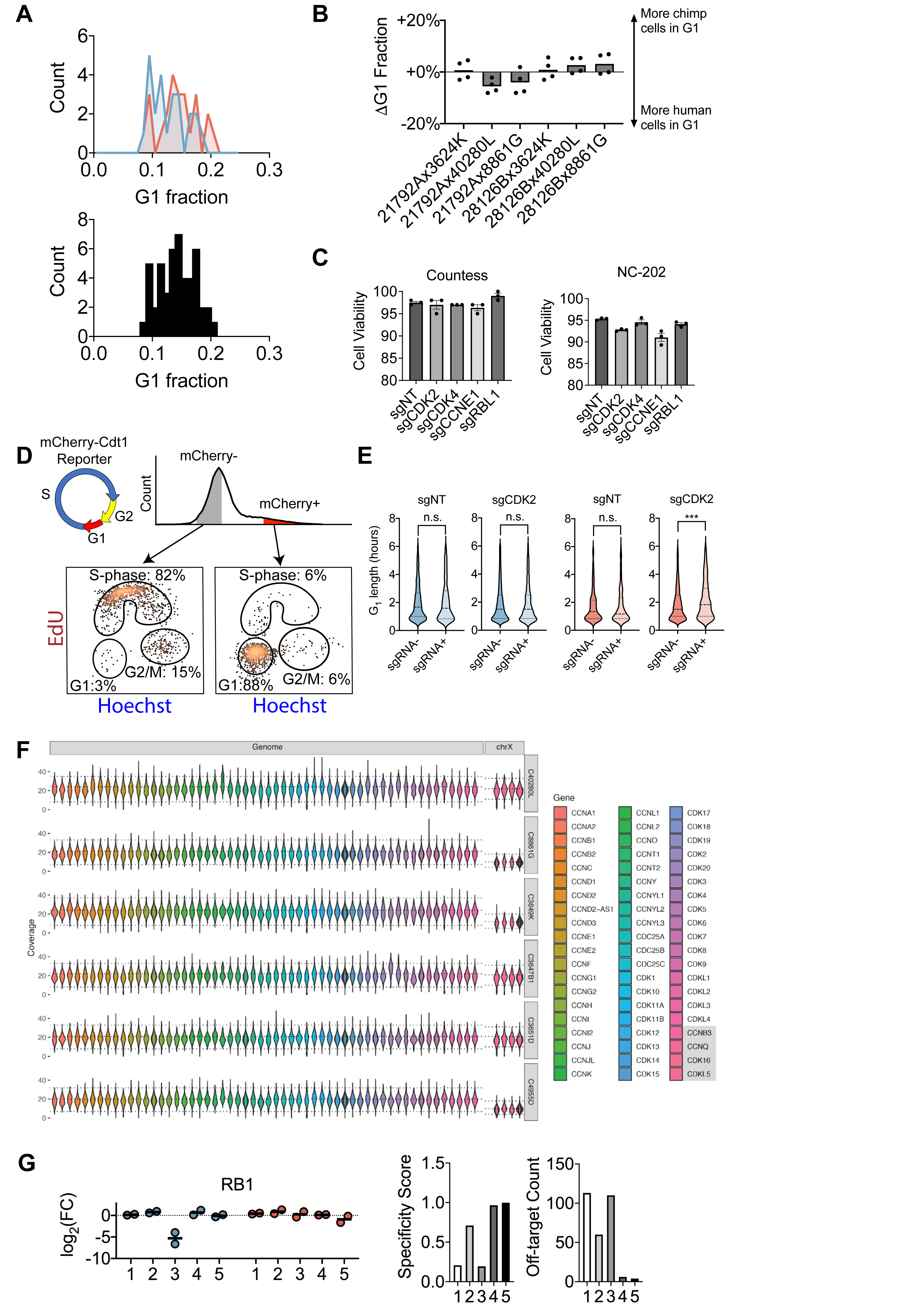

Supplement: Figure S5 — Measurements of species differences in G1 phase length, Related to Figure 4. (A) Absolute fraction of human (21792A and 28126B, blue), chimpanzee (3624K, 40280L, and 8861G, red) PSCs in G1 phase, as measured by EdU incorporation and DNA content. Histogram of G1 fraction for both human and chimpanzee cells (black). (B) Relative fraction of cells in G1 phase for six pairs of human and chimpanzee PSCs co-cultured in the same well (N=4). (C) Cell viability measurements for chimpanzee PSCs (40280L) expressing sgRNAs targeting cell cycle regulators (N=3, s.e.m.). (D) FUCCI reporter cell line cross validation with cell cycle proportion measurements via EdU incorporation and DNA content. (E) Quantification of G1 phase length by live imaging of human (21792A) and chimpanzee (3624K) PSCs infected with either an sgRNA targeting CDK2 or a non-targeting sgRNA. (F) Whole-genome shotgun sequence coverage at all genes in the HUGO Gene Nomenclature Committee gene groups cyclins, cyclin dependent kinases, and class III Cys-based CDC25 phosphatases (https://www.genenames.org/). Each violin represents the coverage at each base across the entire body for each gene. The horizontal lines correspond to the 5th, 50th, and 95th percentiles of baseline coverage across the entire genome (“Genome” panel) or the X chromosome (“chrX” panel). Four genes in these sets located on chromosome X (CDKL5, CDK16, CCNB3, CCNQ) are shown separately to account for different baseline coverage; these gene names are outlined in a gray box in the legend. The top three rows correspond to chimpanzee PSCs from individuals used in the present study (C40280L, C8861G, C3649K), while the bottom three rows correspond to similarly reprogrammed chimpanzee individuals. (G) Strip plots of log2 fold-change for sgRNAs targeting RB1 with data derived from only from primary genome-wide screen. Computationally predicted specificity score and off-target counts for each of the five sgRNAs targeting RB1137. [file NIHMS1920094-supplement-Figure_S5.tif]

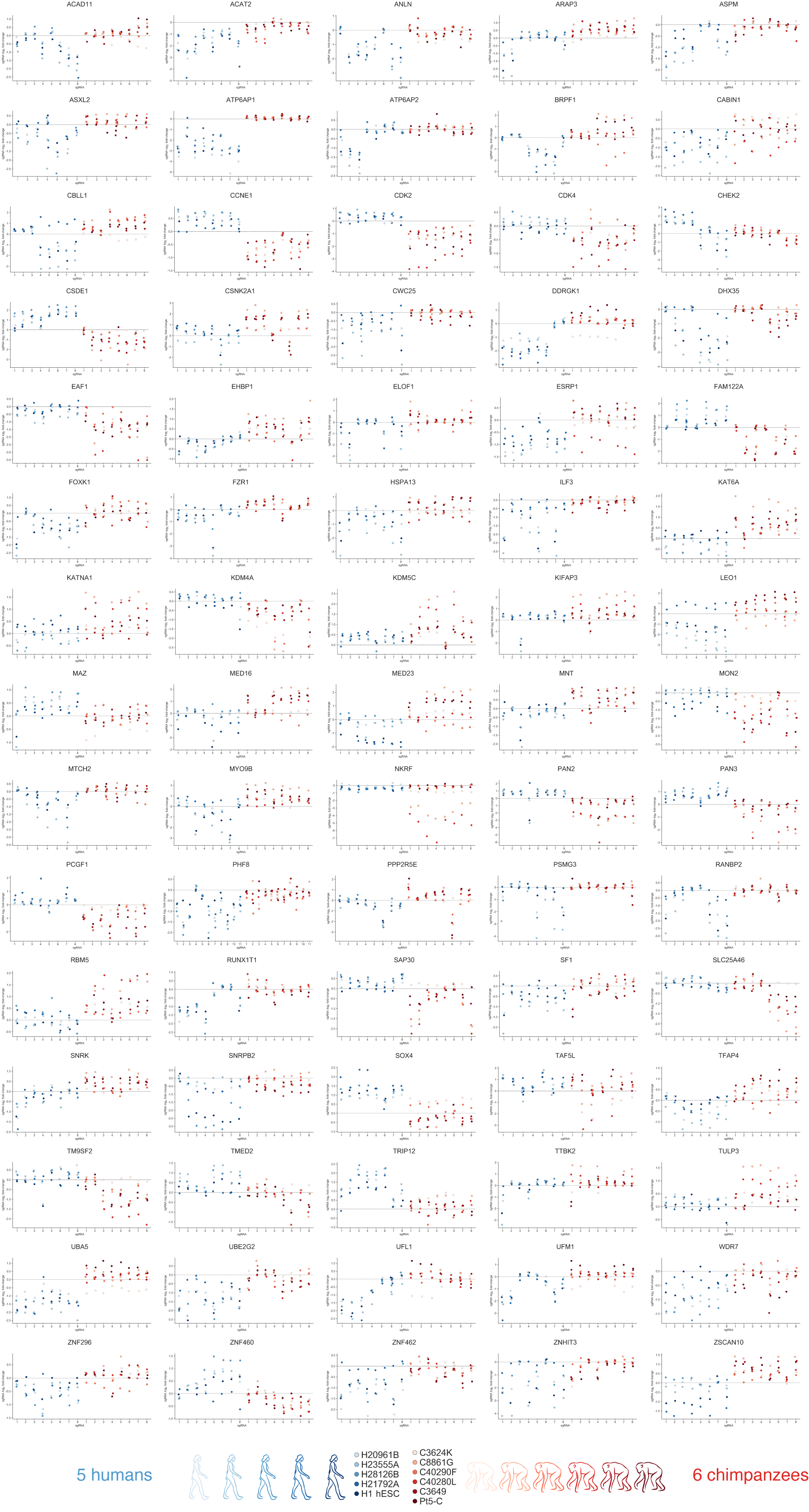

Supplement: Figure S4 — Species-specific genetic dependencies interact in biological processes and complexes, Related to Figure 3. (A) dN, the rate of non-synonymous substitutions in a gene, and (B) dN/dS values, the ratio between the rates of non-synonymous and synonymous substitutions, for 75 validated differential-essentiality genes from this study compared to all genes or essential genes. (C) Comparative gene expression levels between human and chimpanzee PSCs for 75 validated differential-essentiality genes from this study vs. all genes expressed in PSCs. (D) sgRNA depletion or enrichment for all active sgRNAs targeting members of the UFMylation pathway, MOZ histone acetylation complex, RBL1, and the PAN2/3 complex. Each circle represents the sgRNA log2 fold-change for one sgRNA in one human (blue) or chimpanzee (red) individual. Each strip plot contains a variable number of columns, corresponding to the number of significant sgRNAs targeting each gene. Genes with only one significant sgRNA (ING5 and RBL1) are scored as less significant compared to genes with multiple significant sgRNAs and require validation of on-target effects. (E) Co-culture of human PSCs (28126B) and chimpanzee PSCs (40280L) with wild-type cells (BFP−) and cells expressing sgATP6AP1 (BFP+) stained with LysoSensor Green and LysoTracker Red. Scale bar = 20 μm. (F) Western blot for phospho-S6 (pS6) expression and GAPDH loading control for one additional human (21792A) and one chimpanzee (8861G) cell line depleted for ATP6AP1 with a non-targeting sgRNA control. [file NIHMS1920094-supplement-Figure_S4.tif]

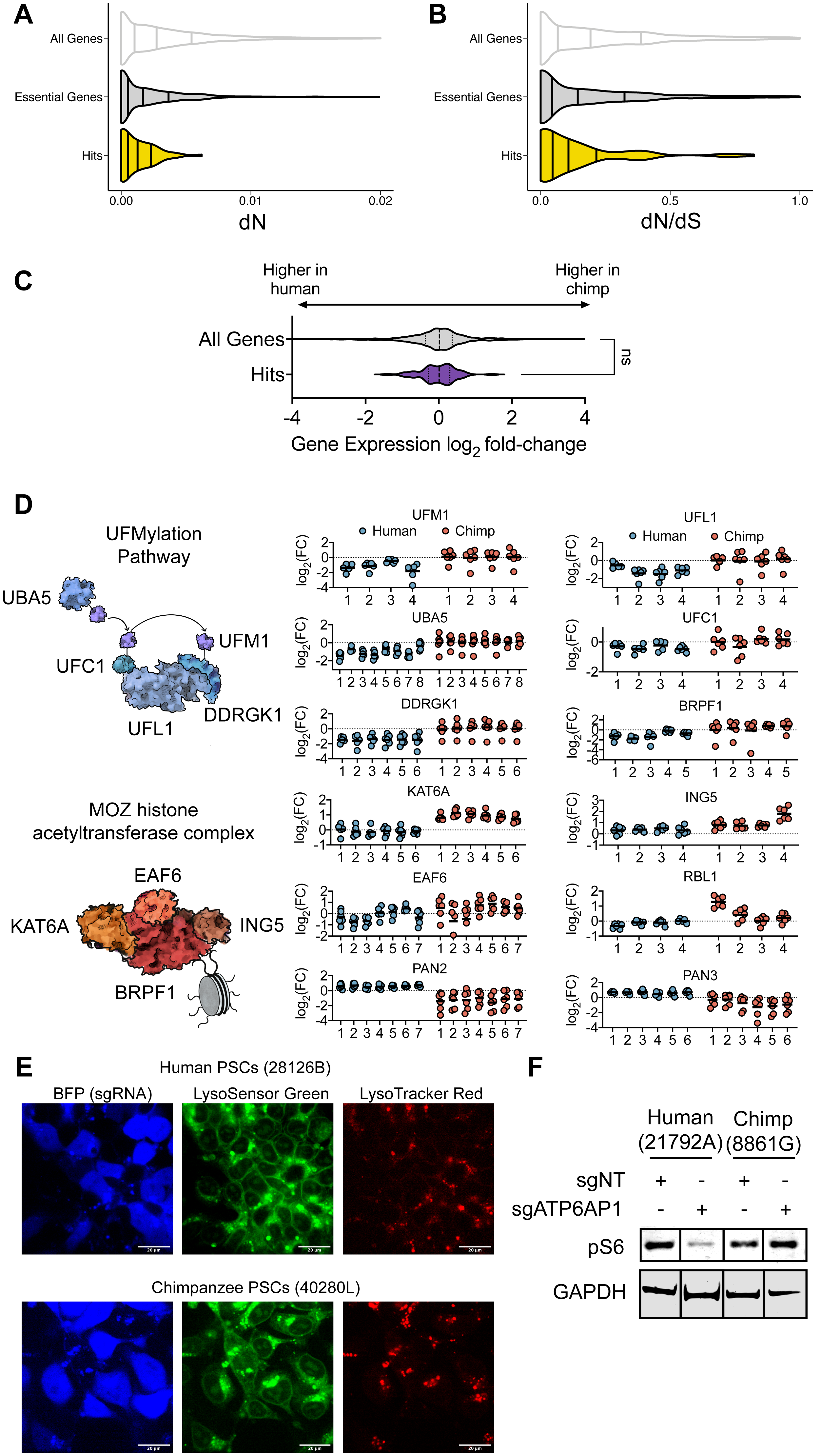

Supplement: Figure S3 — 75 genes with robust species-specific effects on cellular proliferation, Related to Figure 2. (A) Strip plots of log2 fold-change in sgRNA enrichment or depletion for 75 genes with species-specific effects on cellular proliferation (FDR < 1%), calculated by α-RRA, colored by individual. [file NIHMS1920094-supplement-Figure_S3.tif]
